# Genomic Evidence for the Evolution of *Streptococcus equi*: Host Restriction, Increased Virulence, and Genetic Exchange with Human Pathogens

**DOI:** 10.1371/journal.ppat.1000346

**Published:** 2009-03-27

**Authors:** Matthew T. G. Holden, Zoe Heather, Romain Paillot, Karen F. Steward, Katy Webb, Fern Ainslie, Thibaud Jourdan, Nathalie C. Bason, Nancy E. Holroyd, Karen Mungall, Michael A. Quail, Mandy Sanders, Mark Simmonds, David Willey, Karen Brooks, David M. Aanensen, Brian G. Spratt, Keith A. Jolley, Martin C. J. Maiden, Michael Kehoe, Neil Chanter, Stephen D. Bentley, Carl Robinson, Duncan J. Maskell, Julian Parkhill, Andrew S. Waller

**Affiliations:** 1 Wellcome Trust Sanger Institute, Wellcome Trust Genome Campus, Hinxton, Cambridge, United Kingdom; 2 Centre for Preventive Medicine, Animal Health Trust, Lanwades Park, Kentford, Newmarket, Suffolk, United Kingdom; 3 Department of Infectious Disease Epidemiology, Imperial College London, St. Mary's Hospital Campus, London, United Kingdom; 4 The Peter Medawar Building for Pathogen Research and Department of Zoology, University of Oxford, Oxford, United Kingdom; 5 Institute for Cell and Molecular Biosciences, The Medical School, University of Newcastle upon Tyne, Newcastle upon Tyne, United Kingdom; 6 Department of Veterinary Medicine, University of Cambridge, Cambridge, United Kingdom; Children's Hospital Boston, United States of America

## Abstract

The continued evolution of bacterial pathogens has major implications for both human and animal disease, but the exchange of genetic material between host-restricted pathogens is rarely considered. *Streptococcus equi* subspecies *equi* (*S. equi*) is a host-restricted pathogen of horses that has evolved from the zoonotic pathogen *Streptococcus equi* subspecies *zooepidemicus* (*S. zooepidemicus*). These pathogens share approximately 80% genome sequence identity with the important human pathogen *Streptococcus pyogenes*. We sequenced and compared the genomes of *S. equi* 4047 and *S. zooepidemicus* H70 and screened *S. equi* and *S. zooepidemicus* strains from around the world to uncover evidence of the genetic events that have shaped the evolution of the *S. equi* genome and led to its emergence as a host-restricted pathogen. Our analysis provides evidence of functional loss due to mutation and deletion, coupled with pathogenic specialization through the acquisition of bacteriophage encoding a phospholipase A_2_ toxin, and four superantigens, and an integrative conjugative element carrying a novel iron acquisition system with similarity to the high pathogenicity island of *Yersinia pestis*. We also highlight that *S. equi*, *S. zooepidemicus*, and *S. pyogenes* share a common phage pool that enhances cross-species pathogen evolution. We conclude that the complex interplay of functional loss, pathogenic specialization, and genetic exchange between *S. equi*, *S. zooepidemicus*, and *S. pyogenes* continues to influence the evolution of these important streptococci.

## Author Summary

Streptococci colonize a diverse range of animals and tissues, and this association is normally harmless. Occasionally some strains of streptococci have an increased ability to cause disease that is often associated with a reduction in the ability to colonize and the acquisition of new genes, which enable the strain to inhabit a new niche. *S. equi* is the causative agent of strangles, one of the most frequently diagnosed and feared infectious diseases of horses, which is believed to have evolved from the closely related and usually harmless *S. zooepidemicus*. We aim to understand the mechanisms by which *S. equi* causes disease by studying and comparing the genomes of these different strains. Here we identify specific genes that have been lost and gained by *S. equi*, which may have directed its transition from colonizer to invader. Several of the novel genes acquired by *S. equi* have also been identified in strains of the closely related bacterium *S. pyogenes* that are associated with increased morbidity and mortality in humans. Our research highlights the role of genetic exchange in cross-species bacterial evolution and argues that the evolution of human pathogens cannot be considered in isolation.

## Introduction

*Streptococcus equi* subspecies *equi* (*S. equi*) is the causative agent of equine strangles, characterized by abscessation of the lymph nodes of the head and neck. Rupture of abscesses formed in retropharyngeal lymph nodes into the guttural pouches leads to a proportion of horses becoming persistently infected carriers. These carriers transmit the organism to naïve horses and play an important role in disease spread. *S. equi* is believed to have evolved from an ancestral strain of *Streptococcus equi* subspecies *zooepidemicus* (*S. zooepidemicus*) [1],[2], which is associated with a wide variety of diseases in horses and other animals including humans. Both of these organisms belong to the same group of streptococci as the human pathogen *Streptococcus pyogenes*. Previous work has shown that *S. equi* produces four superantigens (SeeH, SeeI, SeeL and SeeM) [3]–[5], two secreted fibronectin-binding proteins (SFS and FNE) [6],[7], a novel M-protein (SeM) [8], an H-factor-binding protein (Se18.9) [9] and a novel non-ribosomal peptide synthesis system [10], but little is known about other factors that influence differences in the virulence of these closely related streptococci.

We determined the complete genome sequence of *S. equi* strain 4047 (*Se*4047), a virulent strain isolated from a horse with strangles in the New Forest, England, in 1990 [11] and *S. zooepidemicus* strain H70 (*Sz*H70), isolated from a nasal swab taken from a healthy Thoroughbred racehorse in Newmarket, England, in 2000 [2]. Using comparative genomic analysis to identify *Se*4047-specific loci, and subsequent screening of *S. equi* and *S. zooepidemicus* strains from around the world, we provide evidence of the genetic events that have shaped the evolution of the *S. equi* genome, and led to its emergence as a host-restricted pathogen.

## Results/Discussion

### General features of the genomes

**Figure 1:**
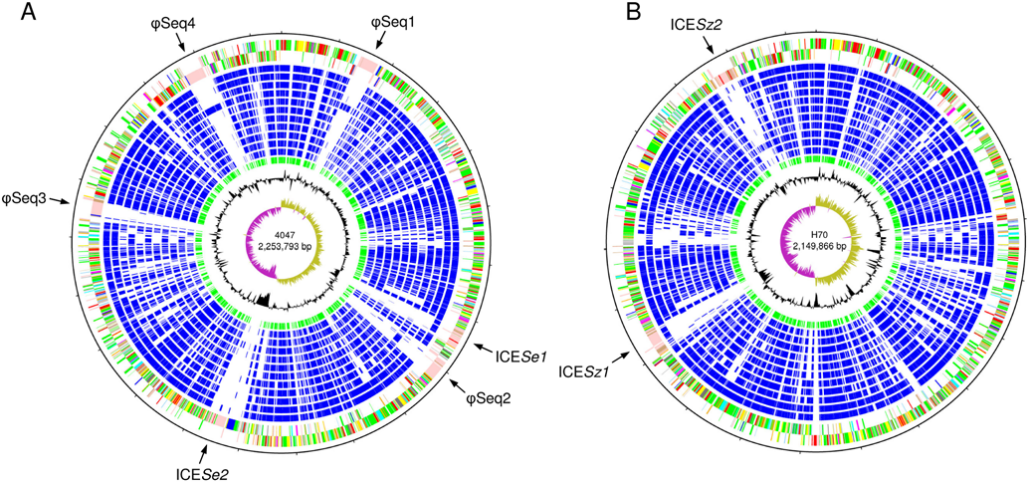
Schematic circular diagrams of the *Se*4047 (A) and *Sz*H70 genomes (B). Key for the circular diagrams (outside to inside): scale (in Mb); annotated CDSs colored according to predicted function represented on a pair of concentric circles, representing both coding strands; orthologue matches shared with the Streptococcal species, *Se*4047 or *Sz*H70, *Sz*MGCS10565, *S. uberis* 0140J, *S. pyogenes* Manfredo, *S.* mutans UA159, *S. gordonii* Challis CH1, *S. sanguinis* SK36, *S. pneumoniae* TIGR4, *S. agalactiae* NEM316, *S. suis* P1/7, *S. thermophilus* CNRZ1066, blue; orthologue matches shared with *Lactococcus lactis* subspecies *lactis*, green; G+C% content plot; G+C deviation plot (>0%, olive, <0%, purple). Color coding for CDS functions: dark blue, pathogenicity/adaptation; black, energy metabolism; red, information transfer; dark green, surface-associated; cyan, degradation of large molecules; magenta, degradation of small molecules; yellow, central/intermediary metabolism; pale green, unknown; pale blue, regulators; orange, conserved hypothetical; brown, pseudogenes; pink, phage and IS elements; grey, miscellaneous. The positions of the four prophage and two ICE*Se* present in the *Se*4047 genome, and two ICE*Sz* in the *Sz*H70 genome, are indicated.

Multilocus sequence typing (MLST) has provided evidence of the close genetic relationship of *S. equi* and *S. zooepidemicus* [2]. The genomes of *Se*4047 (ST-179) and *Sz*H70 (ST-1) support the overall relatedness, but also reveal evidence of genome plasticity that has generated notable diversity. The two genomes are similar in size: the *Se*4047 genome consists of a circular chromosome of 2,253,793 bp (Figure 1A) encoding 2,137 predicted coding sequences (CDSs), and the *Sz*H70 genome contains a chromosome of 2,149,866 bp (Figure 1B), encoding 1,960 predicted CDSs. Much of the *Se*4047 genome is orthologous to the *Sz*H70 genome: 1671 *Se*4047 CDSs have *Sz*H70 orthologs. Of the remaining 466 non-orthologous *Se*4047 CDSs, 422 are found on mobile genetic elements (MGEs; for details of the regions of variation in the *Se*4047 and *Sz*H70 genomes see Table S1).

Recently, the genome sequence of *S. zooepidemicus* strain MGCS10565 (*Sz*MGCS10565) was published [12]. This strain was isolated from a human case of nephritis that was part of a severe epidemic in Brazil [13]. MLST (http://pubmlst.org/szooepidemicus/) analysis indicates that *Sz*H70 and *Sz*MGCS10565 (ST-72) are genetically distinct from each other and *Se*4047. Comparative analysis reveals that the number of orthologs in the *Se*4047 genome is slightly higher for *Sz*H70 (78.2%) than for *Sz*MGCS10565 (77.4%); 76.3% of the *Se*4047 CDSs have matches in both *S. zooepidemicus* strains. For the purposes of this study we have primarily focused our analysis on the comparison of equine isolates, *Se*4047 with *Sz*H70.

**Figure 2:**
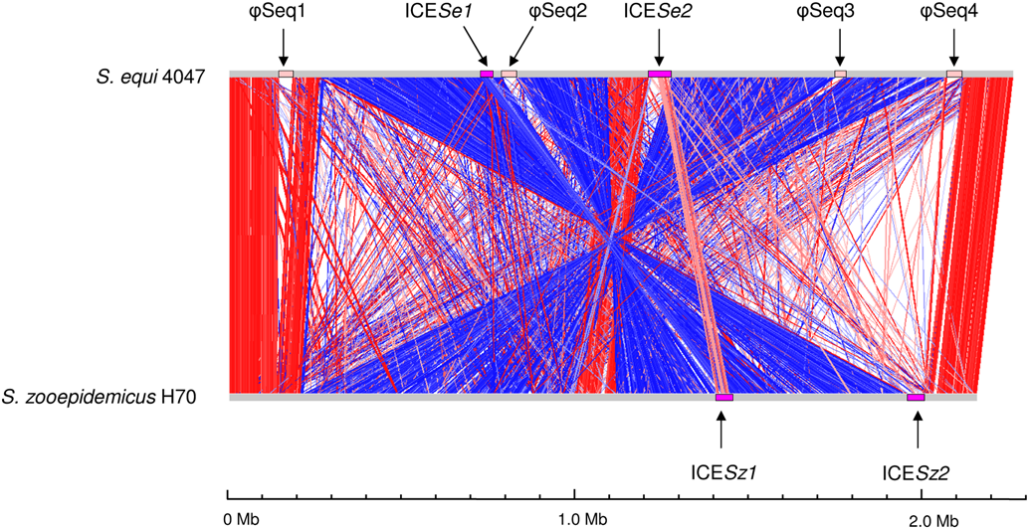
Pairwise comparison of the chromosomes of *Se*4047 and *Sz*H70 using ACT. The sequences have been aligned from the predicted replication origins (*oriC*; right). The colored bars separating each genome (red and blue) represent similarity matches identified by reciprocal TBLASTX analysis [71], with a score cutoff of 100. Red lines link matches in the same orientation; blue lines link matches in the reverse orientation. The prophage (pink) and ICE (purple) are highlighted as colored boxes.

The chromosomes of *Se*4047 and *Sz*H70 are generally collinear except for two inversions around the origin of replication (Figure 2). The smaller central inversion is due to recombination events in *Se*4047 between identical ISSeq3 elements on opposite replichores. The larger rearrangement is due to an inter-replichore inversion in *Sz*H70 of unknown origin (Figure 2). Both the *Se*4047 and *Sz*H70 genomes contain two copies of *hasC* which encode UDP-glucose pyrophosphorylases [14]. In *Sz*H70 one copy of *hasC* (SZO17510) has been translocated to the opposite replichore by the previously mentioned large reciprocal inversion. There is also a small intra-replichore inversion (∼14 kb) in *Se*4047 between the two copies of *hasC* (SEQ0271 and SEQ0289). The *hasC*-mediated inversion in *Se*4047 rearranges the genes associated with capsule production [14] and may explain why *S. equi* produces such high levels of hyaluronate capsule.

**Figure 3:**
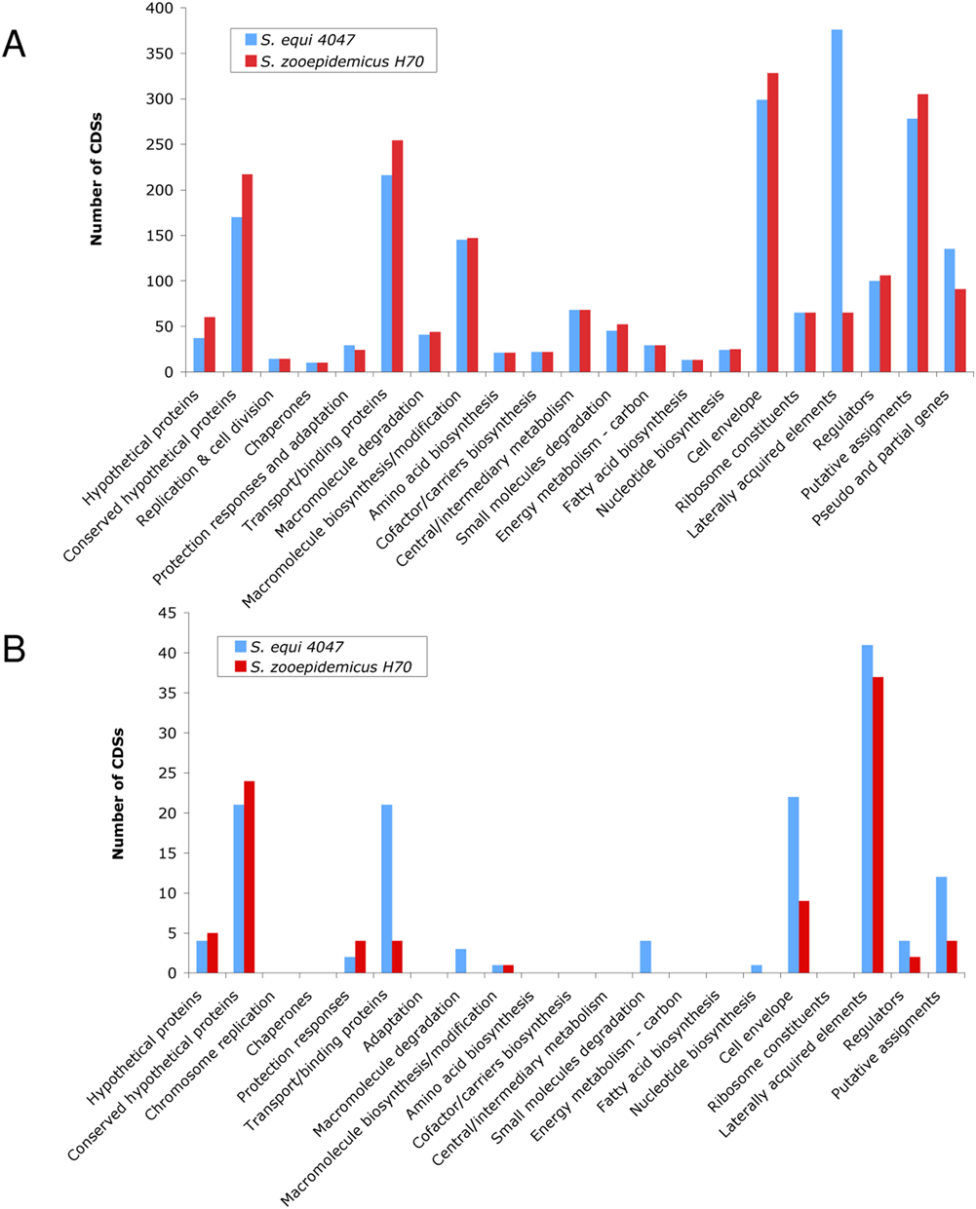
Distribution of CDSs belonging to different functional classes in the *Se*4047 and *Sz*H70 genomes. (A) Functional CDSs and pseudogenes of *Se*4047 and *Sz*H70. (B) Partially deleted or pseudogenes in the *Se*4047 and *Sz*H70 genomes.

Comparison of the predicted functions of the genes encoded in the *Se*4047 and *Sz*H70 genomes revealed that *Se*4047 has the same number, or fewer CDSs, in each of the functional classes with the exception of protective responses and adaptation and laterally acquired elements (Figure 3A). The number of pseudogenes in *Se*4047 is also elevated in comparison to *Sz*H70. The additional protective response and adaptation CDSs in *Se*4047 are associated with the biosynthesis of a putative siderophore [10], and are carried on a MGE region of the genome (ICE*Se2*; Figure 1). The relative expansion of laterally acquired elements, and increased number of pseudogenes in *Se*4047 suggests that the evolution of *S. equi* has been shaped by recent gene loss and gain. A corollary of this genome plasticity appears to have been a reduction in ancestral capabilities, and the introduction of novel functions, which have enabled *S. equi* to exploit a new niche.

### Functional loss

*Se*4047 has 58 partially deleted genes and 78 pseudogenes, compared with 62 and 29 respectively in *Sz*H70 (Figure 3B and Table S1). In particular, *Se*4047 is enriched for mutations associated with catabolic metabolism, transport, and the cell envelope. Such gene loss is typical of other host-restricted bacteria that have evolved from versatile ancestors [15],[16]. The loss of ancestral functions appears to have played a seminal role in the evolution of *S. equi*, resulting in a refinement of its nutritional capabilities, and its host-cell interactions.

**Figure 4:**
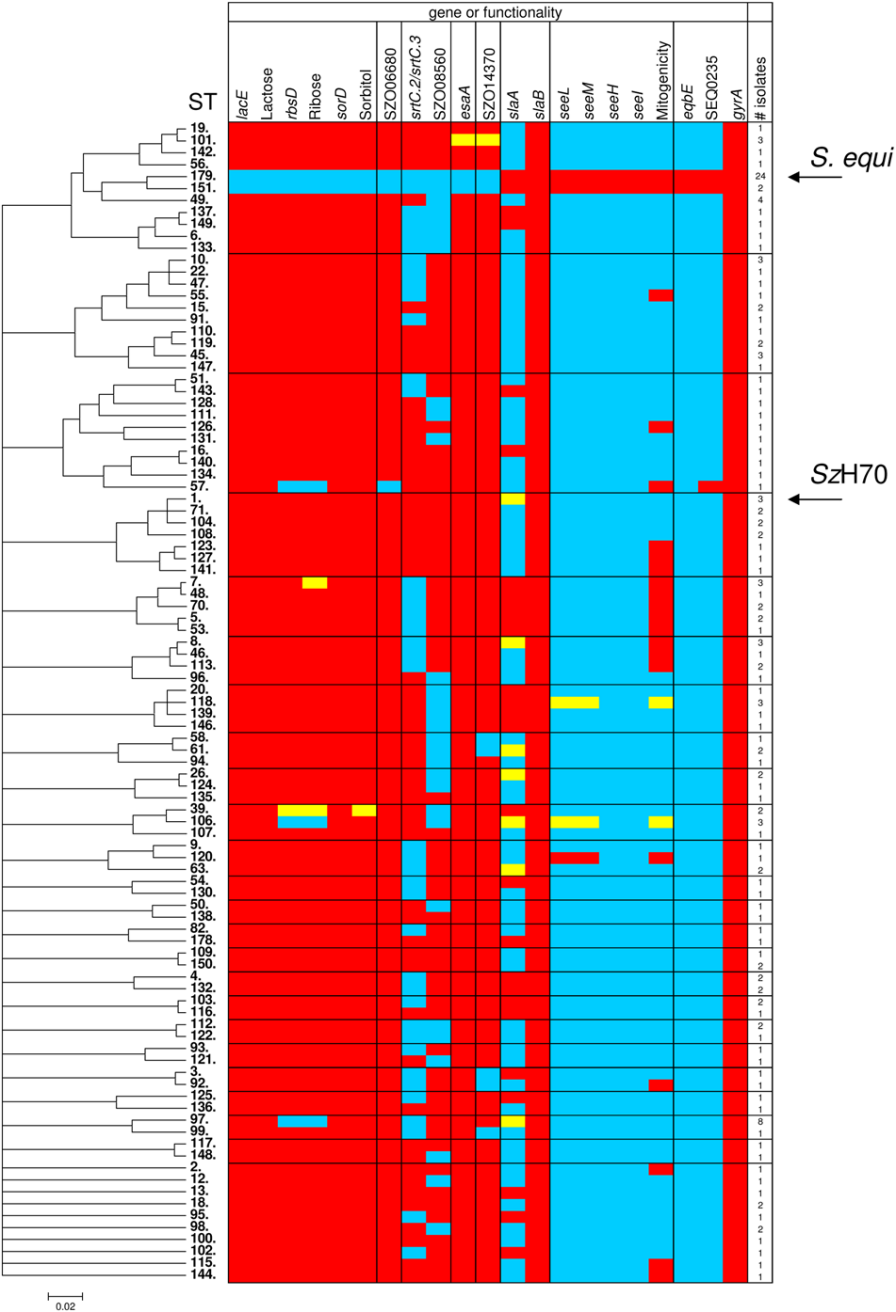
ClonalFrame analysis of MLST alleles of 26 *S. equi* and 140 *S. zooepidemicus* isolates and its relationship with the prevalence of selected differences between the *Se*4047 and *Sz*H70 genomes. Genes examined were *lacE*, *rbsD*, *sorD*, SZO06680 (encoding a putative hyaluronate lyase and specific to the 4 bp missing from SEQ1479), *srtC*, *srtD*, SZO08560 (encoding a *Listeria-Bacteroides* repeat domain containing surface-anchored protein), *esaA*, SZO14370 (within the CRISPR locus), *slaA*, *slaB*, *seeL*, *seeM*, *seeH*, *seeI*, *eqbE* (within the equibactin locus), SEQ0235 (encoding Se18.9), and *gyrA*. Functional assays determined the ability of different isolates to ferment lactose, ribose, and sorbitol and to induce mitogenic responses in equine peripheral blood mononuclear cells. The number of isolates representing each ST is indicated. STs where all isolates contained the gene or possessed functional activity are shown in red, STs where all isolates lacked the gene or functionality are shown in blue, and STs containing some isolates containing the gene or functionality and some that did not are colored in yellow. The position of *S. equi* isolates and *Sz*H70 are indicated. *Sz*MGCS10565 is a single locus variant of ST-10 (ST-72; not shown), and had an identical gene prevalence profile to the ST-10 isolates based on *in silico* analysis of its genome sequence [12].

Carbohydrate metabolism in streptococci plays an important role in colonization of mucosal surfaces [17]. Carbohydrate fermentation is also commonly used to differentiate *S. equi* strains from *S. zooepidemicus* [18]. Comparison of the genome sequences identified a 5 kb deletion in the *Se*4047 genome that partially deleted *lacD* and *lacG* and deleted *lacE*, *lacF* and *lacT*. *Se*4047 also contains a deletion of *sorD* immediately upstream of SEQ0286 and a deletion between SEQ0536 and SEQ0537 that spans the operon required for ribose fermentation. Specialization of *S. equi* has probably rendered these pathways redundant, resulting in their loss. To determine if differences in gene content identified through genome comparison represented variation between *S. equi* and *S. zooepidemicus* or variation within their populations, we screened by PCR a panel of *S. equi* and *S. zooepidemicus* strains that are representative of the wider population as defined by MLST [2]. This included 26 isolates of *S. equi* (representing 2 STs) and 140 isolates of *S. zooepidemicus* (representing 95 STs) [2]. All 26 *S. equi* strains examined lacked *lacE*, *sorD* and *rbsD* and the capacity to ferment lactose, sorbitol or ribose. However, only 15 (ST-7, ST-39, ST-57, ST-97 and ST-106) and 1 (ST-39) of 140 *S. zooepidemicus* isolates tested did not ferment ribose or sorbitol, respectively (Figure 4).

Hyaluronate lyases are secreted enzymes that degrade hyaluronic acid and chondroitins facilitating invasion by bacteria and their toxins [19]. The *Sz*H70 genome contains a single CDS encoding a putative hyaluronate lyase (SZO06680). However, the *Se*4047 orthologue, SEQ1479, contains a 4 bp deletion (TCTC) leading to a frameshift at codon 199. *Se*4047 has acquired a different hyaluronate lyase (SEQ2045) encoded on a prophage. This type of phage-encoded enzyme typically has much lower activity and reduced substrate range [20] than orthologues of SZO06680 [21] and may provide an explanation for why *S. equi* infection rarely progresses beyond the lymphatic system. The 4 bp deleted in strain *Se*4047 was also absent in all 26 strains of *S. equi* tested, whereas these 4 bp were present in all strains of *S. zooepidemicus*. However, one strain of *S. zooepidemicus* (ST-57) was PCR negative due to an IS element insertion 905 bp from the translational start (Figure 4). Reduced hyaluronate lyase activity provides an alternative explanation as to why *S. equi* maintains high levels of hyaluronate capsule and in agreement with this, the ST-57 isolate of *S. zooepidemicus* that tested PCR negative also maintained high levels of capsule. Increased levels of capsule may enhance resistance to phagocytosis [22], but could also reduce adhesion to the mucosal surface [23]. To demonstrate that mucoid colony phenotype was due to hyper-encapsulation, we grew *Se*4047, *Sz*H70 and the ST-57 isolate on plates containing hyaluronidase [24]. All colonies were no longer mucoid in appearance and resembled *Sz*H70 (Figure S1).

Gram-positive bacteria classically display an array of cell wall-anchored proteins on their surface, which are attached covalently through a process mediated by sortase enzymes [25]. In many cases, these cell wall-anchored proteins have been shown to play a role in modulating host-cell interactions. Two putative sortase CDSs are present in the *Se*4047 genome: *srtA* (SEQ1171) and *srtC.1* (SEQ0937), whereas the *Sz*H70 genome contains five: *srtA* (SZO09440), *srtC.1* (SZO11490), *srtC.2* (SZO18270), *srtC.3* (SZO18280) and *srtC.4* (SZO18290). Together, these enzymes potentially process 29 and 39 putative surface proteins in *Se*4047 and *Sz*H70, respectively (Table S2).

*S. equi* strains typically bind significantly lower quantities of fibronectin than those of *S. zooepidemicus* [7]. One possible explanation for this is a one-base deletion within SEQ0375 (*fne*) that was conserved in all strains of *S. equi* examined [7]. The base deletion in *fne* results in the loss of an LPXTG surface anchor and leads to the production of a secreted product, FNE, which binds both fibronectin and collagen [7]. Reduction in the fibronectin-binding properties of *Staphylococcus aureus* increases virulence in a rat pneumonia model [26] and truncation of *fne* has been proposed to increase the virulence of *S. equi* [7]. Our analysis identifies other examples of mutation and gene loss that are likely to contribute to decreased fibronectin binding in *S. equi*. The surface protein Shr of *S. pyogenes* binds heme and transfers it to the streptococcal heme-binding protein Shp for import by the HtsABC heme transporter [27]. Shr also binds fibronectin and contributes to attachment of *S. pyogenes* to epithelial cells [28]. SEQ0443 encoding Shr in *S. equi* contains a frameshift mutation after codon 442 that truncates this protein.

Pili play an important role in the adherence of *S. pyogenes* to host tissues [26]. The *Sz*H70 genome contains two loci that encode genes required putatively for pilus expression. The first of these (SZO11490-SZO11520) shares 84–96% amino acid sequence identity to SEQ0934-SEQ0937 of *Se*4047 and 94–99% amino acid identity with the FimI locus of the recently published human disease isolate *Sz*MGCS10565 [12]. However, the *tetR*-like regulator SEQ0934 of *Se*4047 contains a nonsense mutation at codon 43 that may lead to constitutive pilus production, longer pili that could more effectively protrude through the larger capsule of *S. equi* [29]–[31] and increased collagen-binding [32]. The second *Sz*H70 pilus locus consists of CDSs encoding three putative sortase enzymes, SrtC.2, SrtC.3 and SrtC.4, one putative exported protein (SZ18300) and three putative surface proteins (SZO18310-SZO18330), which share 58%, 76% and 68% amino acid sequence identity with Spy0117, Spy0116 and the fibronectin-binding protein Spy0115 of *S. pyogenes* MGAS10750, respectively [33] and an AraC-like transcriptional regulator (SZO18340). The genome of strain *Se*4047 lacks this putative pilus locus through an ISSeq3 element-mediated deletion. None of the 26 isolates of *S. equi*, but 81 of 140 *S. zooepidemicus* isolates tested positive for *srtC.2* or *srtC.3* by PCR. The genome of *Sz*MGCS10565 does not contain a homologue of this *Sz*H70 pilus locus, but instead contains two other consecutive pilus loci Fim II and Fim III at the same genome location. Fim III is flanked by an AraC-like regulator (Sez_1830), which is orthologous to SZO18340 of *Sz*H70. Diversification of pilus loci could play an important role in the ability of *S. zooepidemicus* strains to infect different hosts and tissues.

**Figure 5:**
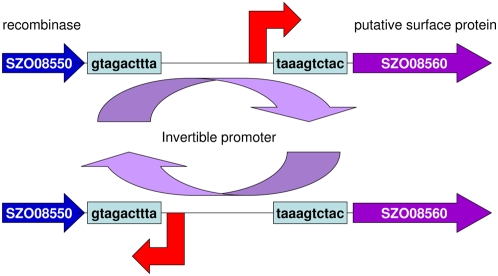
Diagram of the SZO08560 invertible promoter in *Sz*H70. The promoter region of SZO08560 (−170 bp to −55 bp) is bordered by GTAGACTTTA and TAAAGTCTAC inverted repeats that invert to switch transcription from forward to reverse orientation.

The *Sz*H70 and *Sz*MGCS10565 genomes encode a 131 kDa putative surface protein containing 1,160 amino acids with an LPXTG motif (SZO08560 and Sez_1114). However, the *Se*4047 genome encodes only the final 112 amino acids of this protein (SEQ1307a) and lacks an adjacent gene predicted to encode a recombinase (SZO08550 and Sez_1116). SZO08560 and Sez_1114 share sequence similarity with hypothetical proteins of *S. suis* strain 05ZYH33 (SSU05_0473) and *S. agalactiae* strain COH1 (SAN_1519) and contain four *Listeria-Bacteroides* repeat Pfam domains (PF09479). The ∼70 amino acid residue repeats occur in a range of Gram-positive surface proteins including the InlA internalin of *Listeria monocytogenes* [34] (Figure S2). InlA interacts with E-cadherin to promote invasion of *L. monocytogenes* into particular host cells [35]. Examination of the *Sz*H70 genome sequencing data revealed five sequence reads that positioned the promoter region of SZO08560 (−170 bp to −55 bp) in the reverse orientation. This sequence is bordered by GTAGACTTTA and TAAAGTCTAC inverted repeats and we propose that inversion of this sequence switches transcription of SZO08560 on or off, thereby modulating the production of this surface protein in a manner akin to phase variation in *E. coli* (Figure 5) [36]. Reverse transcription qPCR using RNA extracted from log-phase cultures of *Sz*H70 and normalized for expression of the housekeeping gene *gyrA* demonstrated that the SZO08560 promoter of *Sz*H70 transcribed 44-fold more RNA in the forward direction than the reverse. To our knowledge this is the first potential example of recombinase regulation of surface protein production in streptococci. None of the 26 isolates of *S. equi*, but 101 of 140 *S. zooepidemicus* isolates tested positive for SZO08560 by PCR. *Sz*MGCS10565 contains an IS element between the inverted repeats bordering the Sez_1114 promoter and the recombinase (Sez_1116), the consequences of this on transcription of Sez_1114 are not yet known.

Ess (ESAT-6 secretion system) specialized secretion systems have been identified in *Mycobacterium tuberculosis* and *S. aureus* and shown to trigger cell-mediated immune responses including IFN-gamma production that play an important role in virulence [37]. The *Sz*H70 genome contains a cluster of 9 genes (SZO14600-SZO14680) with similarity to the Ess of *Sz*MGCS10565 and *S. aureus* [37]. Unexpectedly, the *Se*4047 genome lacks *esaA*, *esxA* and part of SEQ0576 associated with the presence of an upstream ISSeq3 element (SEQ0575-SEQ0574). PCR analysis showed that none of the 26 strains of *S. equi*, but 138 of 140 *S. zooepidemicus* strains examined contained the *esaA* gene (Figure 4).

### Pathogenic specialization and gene gain

The increased size of the *Se*4047 genome compared to the genome of *Sz*H70 is due to the acquisition of a large number of MGEs. Together these make up a total of 16.4% of the *Se*4047 genome. In contrast 7.5% of the *Sz*H70 genome is composed of MGEs. Several of the MGEs in *Se*4047 carry notable virulence determinants absent in *Sz*H70. The acquisition of these regions by a progenitor may have opened up new pathogenic niches, and been critical in the emergence of *S. equi*.

**Figure 6:**
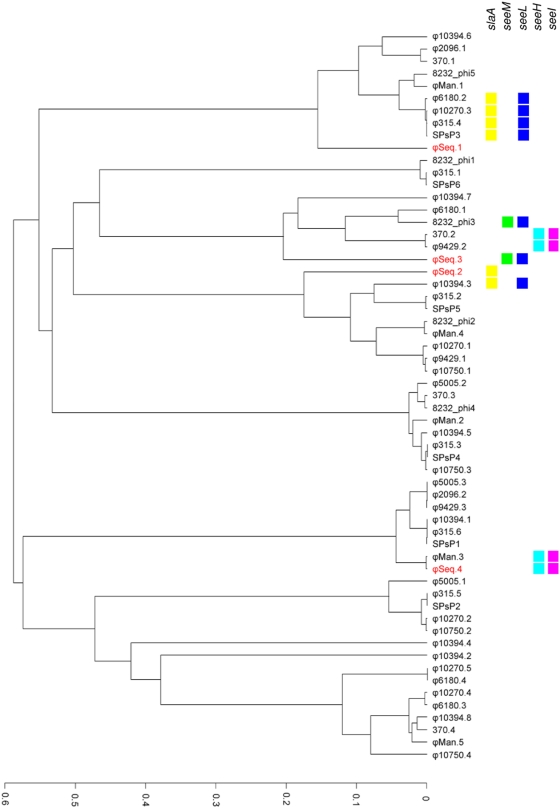
Clustering of *Se*4047 prophage with *S. pyogenes* prophage. UPGMA tree generated from tribeMCL clustering of CDSs from *Se*4047 prophage (highlighted in red) and *S. pyogenes* prophage. *S. pyogenes* prophage used in the clustering were: Manfredo (ϕMan.1, ϕMan.2, ϕMan.3, ϕMan.4, and ϕMan.5), SSI-1 (SPsP1, SPsP2, SPsP3, SPsP4, and SPsP5), SF370 (370.1, 370.2, 370.3, and 370.4), MGAS315 (ϕ315.1, ϕ315.2, ϕ315.3, ϕ315.4, ϕ315.5, and ϕ315.6), MGAS8232 (ϕspeA, ϕspeC, ϕspeL/M, ϕ370.3-like, and ϕsda), MGAS10394 (ϕ10394.1, ϕ10394.2, ϕ10394.3, ϕ10394.4, ϕ10394.5, ϕ10394.6, ϕ10394.7, and ϕ10394.8), MGAS6180 (ϕ6180.1, ϕ6180.2, ϕ6180.3, and ϕ6180.4), MGAS5005 (ϕ5005.1, ϕ5005.2, and ϕ5005.3), MGAS2096 (ϕ2096.1 and ϕ2096.2), MGAS9429 (ϕ9429.1, ϕ9429.2, and ϕ9429.3), MGAS10270 (ϕ10270.1, ϕ10270.2, ϕ10270.3, 10270.4, and 10270.5), and MGAS10750 (ϕ10270.1, ϕ10270.2, ϕ10270.3, and ϕ10270.4). The distribution of homologues to virulence cargo of *Se*4047 prophage are indicated on the right hand side. CDSs belonging to the same homology groups defined using TribeMCL with a cutoff of 1e−5 are indicated by colored blocks: *slaA* (yellow), *seeM* (green), *seeL* (dark blue), *seeH* (light blue), and *seeI* (pink).

Unlike *Sz*H70, *Se*4047 is polylysogenic, containing 4 prophage. The acquisition of prophage plays an important role in the evolution of many pathogenic bacteria [38]. Cargo genes carried by prophage can increase the survival fitness or enhance niche adaptation of the lysogen [38],[39]. Phage repressor and superinfection exclusion functions also confer a selective advantage to the lysogen by providing immunity against lytic infection [40]. Comparison of the sequences of each of the prophage found in *Se*4047 with each other showed only limited mosaic similarity. However, comparison with prophage sequences in the public databases revealed more extensive similarity with prophage from *S. pyogenes*, so much so that clustering analysis has demonstrated that the individual *S. equi* prophage are more related to phage in the other sequenced *S. pyogenes* genomes than they are to each other (Figure 6), suggesting commonality in the phage pool of these pathogens.

The first of the four *Se*4047 prophage, ϕSeq1, is 39 kb in size, contains the CDSs SEQ0133-SEQ0197 and is integrated immediately after the tRNA-Cys gene. The CDSs of ϕSeq1 do not have homology to known virulence factors. However, re-circularized ϕSeq1 was amplified by PCR and confirmed by sequencing across the join of the re-circularized phage following preparation of phage particles present in cultures of *Se*4047 treated with mitomycin C. Release of ϕSeq1 may result in killing of susceptible bacteria, such as *S. zooepidemicus*, which may compete to colonize the epithelium of the equine nasopharynx, thereby resulting in more efficient attachment of *S. equi* and its invasion of the lymphatic system. Such a mechanism is also seen in a lysogen of *Salmonella enterica* serovar Typhimurium, which releases low titers of phage that lysed competing non-lysogenic strains [40].

The 41 kb ϕSeq2 (SEQ0787-SEQ0851) is integrated into the putative C-terminal sequence of an ATP-dependent DNA helicase (SEQ0786) and contains a CDS (SEQ0849) that shares 98% predicted amino acid sequence identity with the phospholipase A_2_, SlaA, of *S. pyogenes* M3 MGAS315 [38]. SlaA is known to contribute to streptococcal virulence and its recent acquisition by *S. pyogenes* M3 (in approximately 1987) was associated with increased morbidity and mortality [38]. Deletion of *slaA* reduced the virulence of *S. pyogenes* in a mouse intraperitoneal infection model and severely compromised its ability to colonize the upper respiratory tract of a macaque model of pharyngitis [41]. *slaA* is widely distributed amongst *S. zooepidemicus* STs (44 of 140 isolates) and all 26 strains of *S. equi* tested here (Figure 4). Of particular note was the observation that *S. equi* CF32, which was isolated from a horse with strangles during 1981, contained *slaA*. This isolate predates all *slaA* positive isolates of *S. pyogenes* [42], and it is possible that *slaA* in the *S. pyogenes* gene pool gene may have arisen via phage-mediated horizontal transfer from a *slaA*-containing strain of *S. zooepidemicus* or *S. equi*, although the precise evolutionary origins remain unclear. We were unable to detect re-circularized ϕSeq2 in phage particle preparations of *Se*4047 following mitomycin C treatment. However, this prophage appears to be intact and could re-circularize in response to other stimuli such as UV irradiation or heat shock. In support of the important role that these toxins may play in colonization and virulence of *S. equi* and *S. zooepidemicus*, we identified a gene encoding a second putative phospholipase A_2_ toxin, SlaB, sharing 70% amino acid sequence identity with SlaA of *S. pyogenes* in the genomes of *Se*4047 (SEQ2155) and *Sz*H70 (SZO18670). This gene, also identified in *Sz*MGCS10565 (Sez_1876), was associated with the remnants of a hypothetical prophage gene and was present in all strains of *S. equi* and *S. zooepidemicus* tested (Figure 4).

The 30 kb ϕSeq3 is integrated into SEQ1725 which encodes a putative late competence protein and contains CDSs SEQ1727-SEQ1765 including two cargo CDSs encoding the superantigens SeeL and SeeM, which share 97% and 96% amino acid sequence identity with SpeL and SpeM of *S. pyogenes* MGAS8232, respectively [3],[5]. The genes encoding SeeL and SeeM were present in all strains of *S. equi* and 4 of 140 isolates of *S. zooepidemicus* tested (Figure 4). Interestingly, these *S. zooepidemicus* isolates represented 3 unrelated STs (ST-106, ST-118 and ST-120) recovered from the same outbreak of equine respiratory disease in 1996. *S. equi* CF32 also contained these superantigen genes, and predates SpeL- and SpeM-producing strains of *S. pyogenes* [43], providing further evidence that *S. equi* and *S. zooepidemicus* act as reservoirs of virulence genes that may be transferred by lateral gene transfer events. Re-circularized ϕSeq3 was not detected by PCR of mitomycin C induced phage particle preparations of *Se*4047. However, the CDSs of this prophage appear to be intact and may permit re-circularization in response to other stimuli.

**Figure 7:**
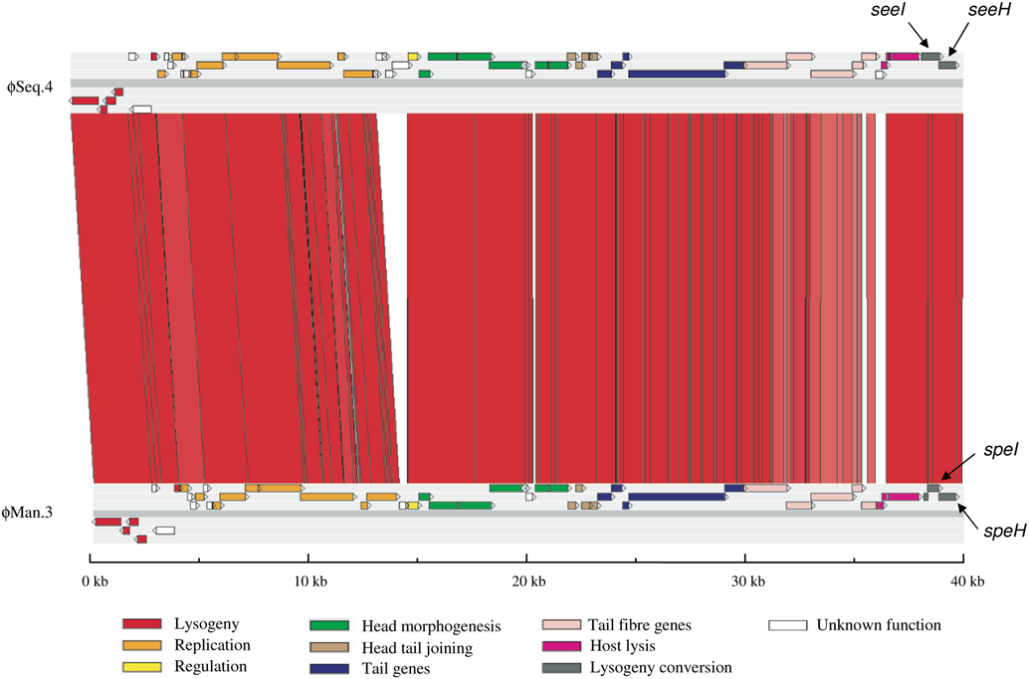
Pairwise comparison of *Se*4047 ϕSeq.4 and ϕMan.3 from *S. pyogenes* Manfredo displayed using ACT. The red bars separating each sequence represent similarity matches identified by TBLASTX analysis. The locations of *seeI*, *seeH*, *speI*, and *speH* are indicated.

Finally, the 40 kb ϕSeq4 is inserted next to SEQ2035, resulting in the truncation of this putative transcriptional repressor. ϕSeq4 contains cargo CDSs encoding the previously described superantigens SeeH (SEQ2036) and SeeI (SEQ2037), which share 98% and 99% amino acid sequence identity with SpeH and SpeI, respectively [4]. Interestingly, ϕSeq4 was very closely related to ϕMan3 of *S. pyogenes* Manfredo (Figure 7). Although *seeH* and *seeI* were present in all strains of *S. equi* tested, we have not yet identified any strains of *S. zooepidemicus* that contain these genes. However, re-circularized ϕSeq4 was detected by PCR and confirmed by sequencing across the join of the re-circularized phage purified from cultures of *Se*4047 treated with mitomycin C. Our data suggest that the acquisition of ϕSeq4 by *S. equi*, possibly originating from a strain of *S. pyogenes*, may have been a very recent event that could have influenced the emergence of *S. equi*.

To provide functional evidence for the production of superantigens by different strains of *S. equi* and *S. zooepidemicus*, we assayed the culture supernatants of our strain collection. This confirmed that all strains of *S. equi* and the strains of *S. zooepidemicus* containing *seeL* and *seeM* possessed significant mitogenic activity. However, the supernatants of 25 additional strains of *S. zooepidemicus* also had mitogenic activity. Several of these strains were related genetically by MLST, and clustered into three groups (ST-123, ST-127 and ST-141; ST-7, ST-48, ST-70, ST-5 and ST-53; ST-8, ST-46 and ST-113) (Figure 4 and Table S3). We propose that these strains probably contain genes encoding other *S. pyogenes* superantigens or novel genes that represent an additional reservoir of as yet uncharacterized superantigens.

The absence of prophage in the *Sz*H70 genome, and low frequency of phage associated superantigens in the screening of *S. zooepidemicus* strains, is in stark contrast to *S. equi*. One explanation for the lack of prophage in *S. zooepidemicus* is that systems exist in naturally transformable streptococci that provide resistance to uptake and incorporation of foreign DNA and may co-incidentally prevent stable prophage integration [12]. *Se*4047 lacks 9 putative competence genes (Table S1) that are intact in *Sz*H70 and *Sz*MGCS10565, which could provide an explanation for the polylysogenic nature of *Se*4047. An alternative explanation of the proliferation of prophage in *S. equi* can be found in the genome comparison between *Sz*H70 and *Se*4047. In the *Sz*H70 genome a locus containing a clustered regularly interspaced short palindromic repeat (CRISPR) array and CRISPR-associated (CAS) genes (SZO14370-SZO14430) was identified, which has been deleted from the *Se*4047 genome due to recombination between ISSeq11 elements (Table S1). CRISPR arrays are composed of direct repeats that are separated by similarly-sized non-repetitive spacers. These arrays, together with a group of associated proteins, confer resistance to phage directed by sequence similarity between the spacer regions and the phage in question, possibly via an RNA-interference-like mechanism [44],[45]. The *Sz*H70 CRISPR contains eighteen spacer sequences, of which ten have no significant database matches, three share >94% identity with prophage sequences present in the published genomes of *S. pyogenes*, four spacers have identical matches with prophage sequences found in the *Se*4047 genome (#6 with SEQ0163, #7 with SEQ1743, #8 with SEQ1745 and #15 with SEQ1727 (*seeM*)) and one spacer (#18) has a near identical match with the *Se*4047 prophage CDS SEQ0190, differing only at the first nucleotide (C to T). This latter spacer is the only exact match with the spacer sequences of *Sz*MGCS10565 CRISPRs (spacer 9 of CRISPR I) [12]. The limited spacer similarity of *Sz*H70 and *Sz*MGCS10565 may reflect exposure to different phage in their respective host environments.

The CRISPR loci of *Sz*H70 and *Sz*MGCS10565 may assist the development of resistance to circulating phage and maintain genome integrity. The CRISPR region of *Sz*H70 was present in 93% (131/140) of *S. zooepidemicus* isolates examined by PCR, but was absent from all strains of *S. equi* tested (Figure 4). Deletion of the CRISPR locus from the ancestor of *Se*4047 is likely to have resulted in increased genome instability and illustrates that in some circumstances gene loss may in turn influence the subsequent rate of gene gain.

Both the *Sz*H70 and *Se*4047 genomes contain distinct integrative conjugative element (ICE) regions. This type of MGE element has been shown to be widely distributed [46], and associated with the transfer of a diverse range of functions. One of the ICE in the *Se*4047 genome, ICE*Se2*, contained CDSs (SEQ1233-SEQ1246) with similarity to the non-ribosomal peptide synthesis (NRPS) system of *Clostridium kluyveri* and *Yersinia* sp. that produce an unnamed siderophore [47] and the ferric iron-binding siderophore yersiniabactin [48], respectively. We have demonstrated that the *S. equi* NRPS operon is required for the production of an undefined secreted molecule, provisionally named equibactin, which enhances the ability of *S. equi* to acquire iron [10]. Siderophore biosynthesis has not previously been identified in any streptococci [49]. However, homologues of SEQ1246 and SEQ1243 (present as a pseudogene) are in the genome of *S. agalactiae* NEM316 serotype III, suggesting that a locus with similarity to the *S. equi* NRPS operon may have been important to this organism at some time.

The ICE*Se2* locus was present in all of the *S. equi* isolates, but in none of the diverse collection of *S. zooepidemicus* isolates examined (Figure 4). Given the importance of iron acquisition to other streptococcal pathogens [50], the acquisition of ICE*Se2* may have contributed significantly to the increased pathogenesis of this *Streptococcus*. In particular, we hypothesize that more efficient acquisition of iron could enhance the ability of *S. equi* to generate lymph node abscessation, which is critical to the establishment of long term carriage and vital to the success of this bacterium. It is intriguing to note that the production of yersiniabactin by *Y. pestis* is essential to its virulence [51]. It will be important to determine the contribution of ICE*Se2* to the formation of abscesses in the lymph nodes of horses.

A facet of the *Se*4047 genome suggestive of recent niche adaptation is the large increase in the number of IS elements relative to *Sz*H70 (*Sz*H70 contains 30 whereas *Se*4047 contains 73; Table S4). In particular there appears to have been an expansion of the IS3-family IS element, ISSeq3: the *Se*4047 genome contains 40 copies of ISSeq3 whereas *Sz*H70 contains 4 (ISSzo3). An expansion of IS elements has been observed in several host-restricted pathogens, which have recently evolved from generalist ancestors [15],[16]. An evolutionary consequence of niche transit is hypothesized to be that many genes become dispensable, allowing increased inactivation. Niche change is also associated with significant evolutionary bottlenecks, which will be enhanced by repeated acquisition of mobile genetic elements. This leads to small effective population sizes, resulting in lower efficiency of selection, which in turn allows gene mutation and expansion of IS elements through accelerated genetic drift. A corollary of the IS proliferation has been the loss of genes by deletion [15]: several of the previously described examples of gene loss (eg. pilus locus and CRISPR locus) probably occurred through insertion and recombination between IS elements (Table S1).

### Conclusions

**Figure 8:**
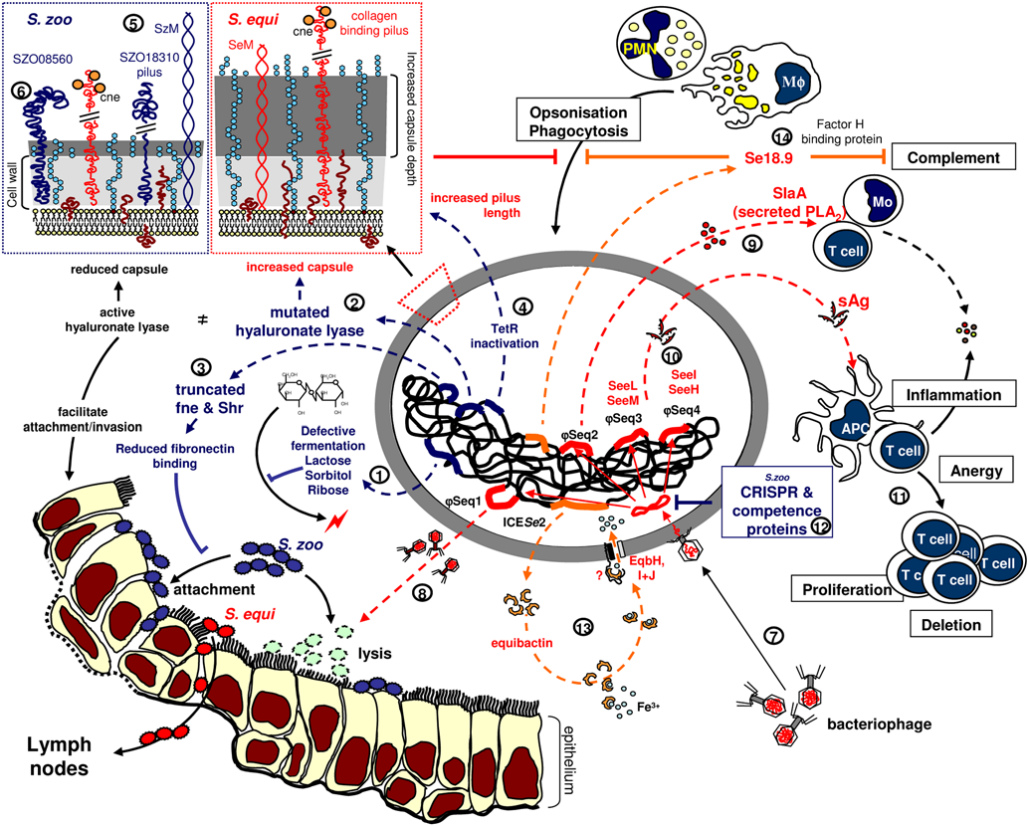
Summary of functional loss and gene gain by *S. equi*. Gene loss (blue): (1) *Se*4047 has lost the ability to ferment lactose, sorbitol, and ribose, which may reduce its ability to colonize the mucosal surface. (2) Hyaluronate lyase activity is predicted to be reduced in *Se*4047, which could decrease its ability to invade tissue and provide an explanation for increased levels of hyaluronate capsule. Increased levels of capsule may enhance resistance to phagocytosis, but could also reduce adhesion to the mucosal surface. (3) Truncation of fne and Shr in *Se*4047 and subsequent synthesis of secreted fibronectin products may decrease the adhesive properties of *Se*4047 and interfere with fibronectin-dependent attachment mechanisms of competing pathogens. (4) Loss of function of the *tetR* regulator may lead to constitutive production of longer collagen-binding pili by *S. equi*. (5) The putative SZO18310 pilus locus of *Sz*H70 has been deleted from the *Se*4047 genome. (6) *Se*4047 has lost a *Listeria-Bacteroides* repeat domain containing surface-anchored protein. Gene gain (red): (7) The acquisition of prophage plays an important evolutionary role through integration of cargo genes. (8) Recirculation and secretion of the integrated ϕSeq1 may kill susceptible competing bacteria such as *S. zooepidemicus*. (9) ϕSeq2 contains a gene encoding a phospholipase A_2_ (SlaA) that may enhance virulence. (10) ϕSeq3 and ϕSeq4 encode superantigens SeeH, SeeI, SeeL, and SeeM that target the equine immune system (11). (12) The absence of prophage in *S. zooepidemicus* may be explained by the presence of CRISPR arrays and competence proteins that confer resistance to circulating phage and maintain genome integrity. (13) The ICE*Se*2 locus may enhance iron acquisition in *Se*4047 through the production of a potential siderophore, equibactin. (14) Se18.9 binds Factor H and interferes with complement activation.

The comparison of the genomes of *Se*4047 and *Sz*H70 provides strong evidence that *S. equi* has passed through a genetic bottleneck during its evolution from an ancestral *S. zooepidemicus* strain. We have identified several examples of gene loss that serve to reduce the ancestral capabilities of *S. equi* and increase the opportunity for genetic change. The acquisition of new mobile genetic elements has been critical to the evolution of *S. equi*. However, surveillance of the *S. zooepidemicus* population has identified examples of strains that did not cause strangles, but contain genes encoding phospholipase A_2_ toxins and superantigens. Therefore, we propose that the key speciation event in the evolution of *S. equi* was the acquisition of ICE*Se2*, containing a novel NRPS involved in the acquisition of iron, which is the first of its kind to be identified in streptococci. The proposed functional effects that result from the genetic events highlighted by our analysis are summarized in Figure 8.

Our study provides strong evidence for genetic exchange between *S. equi*, *S. zooepidemicus* and *S. pyogenes*, which continues to influence the pathogenicity of these important bacteria. The genetic diversity of the *S. zooepidemicus* population as measured by MLST [2] suggests that further investigation of this species will be likely to identify many more genes of importance to both veterinary and human disease.

## Materials and Methods

### Strains growth and DNA isolation

*Se*4047 was isolated from a horse with strangles in the New Forest, England, in 1990 [11], and has been typed as ST-179 by MLST [2]. *Sz*H70 was isolated from a nasal swab taken from a healthy Thoroughbred racehorse in Newmarket, England, in 2000, and has been typed as ST-1 by MLST [2]. Details of all of the isolates examined in this study are presented in Table S3 and are also available on the online MLST database (Available: http://pubmlst.org/szooepidemicus/. Accessed 3 October 2008).

For the preparation of DNA for whole genome sequencing *Se*4047 and *Sz*H70 were grown overnight in Todd Hewitt broth (THB) at 37°C in a 5% CO_2_ enriched atmosphere. Cells were harvested and chromosomal DNA was extracted according to the method of Marmur [52] with the addition of 5000 units of mutanolysin (Sigma) and 20 µg of RNaseA (Sigma) during the lysis step.

For the study of hyaluronate capsule degradation strains were grown overnight on COBA strep select plates (bioMérieux) at 37°C in a 5% CO_2_ enriched atmosphere, with and without pre-absorption of plates with 50 µl of 40 mg ml^−1^ hyaluronidase (Sigma cat# H2126).

### Whole genome sequencing

The genome of *Se*4047 was obtained with ∼8× coverage from m13mp18 and pUC18 genomic shotgun libraries (with insert sizes of 1.4 to 4 kb) using big-dye terminator chemistry on ABI3700 automated sequencers. Large insert BAC libraries (pBACe3.6, with insert sizes of 10–20 kb; and pEpiFos1, with insert sizes of 38–42 kb) were used as scaffolds. The *Sz*H70 genome was obtained with ∼8× coverage from pUC18 and pMAQ1b genomic shotgun libraries (with insert sizes of 2–6 kb) using big-dye terminator chemistry on ABI3700 automated sequencers. A large insert pBACe3.6 library (with insert sizes of 20–23 kb) was used as a scaffold. Repeats were bridged by read-pairs or end-sequenced PCR products.

### Annotation and analysis

The sequence was finished and annotated as described previously using Artemis software to collate data and facilitate annotation [53]. Comparison of the genome sequences was facilitated by using the Artemis Comparison Tool (ACT) [54]. Orthologous proteins were identified as reciprocal best matches using FASTA [55] with subsequent manual curation. Orthology inferred from positional information was investigated using ACT. Pseudogenes had one or more mutations that would prevent correct translation; each of the inactivating mutations was subsequently checked against the original sequencing data. The sequence and annotation of the *Se*4047 and *Sz*H70 genomes have been deposited in the EMBL database under accession numbers FM204883 and FM204884 respectively.

Sequences used for comparative genomic analysis were: *S. zooepidemicus* MGCS10565 (CP001129) [12], *S. uberis* 0140J (AM946015) [56], *S. pyogenes* Manfredo (AM295007) [57], *S. thermophilus* CNRZ1066 (CP000024) [58], *S. suis* P1/7 (http://www.sanger.ac.uk/Projects/S_suis/) (Holden *et al.*, unpublished), *S. pneumoniae* TIGR4 (AE005672) [59], *S. sanguinis* SK36 (CP000387) [60], *S. mutans* UA159 (AE014133) [61], *S. agalactiae* NEM316 (AL732656) [62], *S. gordonii* str. Challis substr. CH1 (CP000725) [63] and *Lactococcus lactis* subsp. *lactis* IL1403 (AE005176) [64].

### Prophage Clustering

*S. pyogenes* prophage sequences were extracted from the genomes of *S. pyogenes* strains Manfredo (AM295007) [57]; SSI-1 (BA000034) [65], SF370 (AE004092) [66], MGAS315 (AE014074) [42], MGAS8232 (AE009949) [59], MGAS10394 (CP000003) [60], MGAS6180 (CP000056) [61], MGAS5005 (CP000017) [67], MGAS2096 (CP000261) [33], MGAS9429 (CP000259) [33], MGAS10270 (CP000260) [33] and MGAS10750 (CP000262) [33]. Prophage CDSs were clustered into homology groups using TribeMCL (Centre for Mathematics and Computer Science and EMBL-EBI) [68] with a cut-off of 1e−50.

### Sugar fermentation

The ability of isolates to ferment lactose, ribose and sorbitol was determined in Purple broth (Becton Dickinson) as previously described [18].

### Mitogenicity assays

Equine PBMC were purified from heparinised blood by centrifugation on a Ficoll density gradient. PBMC were incubated with *S. equi* or *S. zooepidemicus* culture supernatants diluted 1/20. PBMC proliferation was detected by overnight incorporation of ^3^H thymidine after 3 days of culture. Equine PBMC proliferation is expressed as stimulation index (SI) calculated as follows (experimental response/control response). A SI≥2 was considered as positive.

### Gene prevalence studies

Genomic DNA from a diverse set of 26 *S. equi* strains and 140 *S. zooepidemicus* strains was prepared from single colonies grown on COBA strep select plates (bioMérieux) and purified using GenElute spin columns according to manufacturer's instructions (Sigma). The relatedness of MLST STs was determined using ClonalFrame [69]. Gene prevalence was then determined by quantitative PCR (QPCR) using a SYBR green based method with a Techne Quantica instrument. For the QPCR, 10 ng DNA diluted was mixed with 0.3 µM forward and reverse primers (Table S5) and 1× ABsolute QPCR SYBR green mix (Abgene) in a total volume of 20 µl and subjected to thermocycling at 95°C for 15 min, followed by 40 cycles of 95°C for 15 s, 55°C for 30 s and 72°C for 30 s. Dissociation curves were analyzed following a final ramp step from 60°C to 90°C with reads at 0.5°C increments to rule out non-specific amplification. Data were analyzed using Quansoft software (Techne). Crossing point values relative to those for the *gyrA* house-keeping gene were used to determine gene presence or absence.

### Reverse transcription and quantitative real-time PCR for recombinase activity

The potential for inversion of the promoter region proceeding the recombinase was assessed by comparison of SZO08560 mRNA transcript levels (produced when the promoter region is in the forward orientation) with reverse strand SZO08550 mRNA transcript levels (produced when the promoter region is inverted) in *Sz*H70. *Sz*H70 was grown to log phase in THB with 10% horse sera. A quantitative two-step reverse transcription (RT) PCR procedure was used to analyze levels of SZO08560 and reverse strand SZO08550 transcription relative to the housekeeping gene *gyrA*. RT was performed using the Verso cDNA kit (Abgene). The RT reaction mixture (20 µl) contained 100 ng total RNA, 2 µM gene-specific primer (ZM474R or ZM476F) (Table S5), 500 µM dNTP mix, 1× cDNA synthesis buffer, 1 µl RT enhancer and 1 µl Verso enzyme mix. RT was performed at 50°C for 30 min and terminated by heating to 95°C for 2 min. Quantitative real time PCR (QPCR) was performed with a Techne Quantica instrument and data analyzed using Quansoft software (Techne). For the QPCR, 6 µl RT reaction mixture diluted 1/1000 was mixed with 0.3 µM forward and reverse primers (Table S5), and 1× ABsolute QPCR SYBR green mix (Abgene) in a total volume of 20 µl and subjected to thermocycling at 95°C for 15 min, followed by 40 cycles of 95°C for 15 s, 55°C for 30 s and 72°C for 30 s. Dissociation curves were analyzed, following a final ramp step from 60°C to 90°C with reads at 0.5°C increments, to rule out non-specific amplification. No-template negative controls were included and reverse transcriptase negative controls to confirm the absence of contaminating DNA from RNA samples. Standard curves (Crossing point vs. log gene copy number) were generated from genomic DNA for each target gene and used to calculate transcript copy number in cDNA samples. SZO08560 and reverse strand SZO08550 transcript copy numbers were normalized to *gyrA* reference gene copy number to correct for differences in the amount of starting material. Data was expressed as fold difference in normalized SZO08560 transcript level relative to reverse strand SZO08550 transcript level.

### Phage particle DNA purification, PCR, and sequencing

Phage particle DNA was purified according to previously published methods [70]. *Se*4047 was grown to log phase and treated for 3 hours with mitomycin C. Bacteria were centrifuged at 8,000×g for 15 minutes and the supernatant was sterilized with a 0.45 µm filter (Millipore). The filter-sterilized supernatant was centrifuged at 141,000×g for 4 h at 10°C, and the pellet resuspended in 1 ml phage suspension buffer. 0.5 ml phage particles were treated with 25 U benzonase (Novagen) for 1 h at 37°C and then lysed with 0.5% sodium dodecyl sulfate, 10 mM EDTA and 500 µg of proteinase K (Sigma)/ml for 1 h at 37°C. Phage DNA was extracted with an equal volume of phenol-chloroform-isoamyl alcohol (25∶24∶1) (Sigma), followed by an equal volume of chloroform-isoamyl alcohol (24∶1) (Sigma). Phage DNA was precipitated with 300 mM NaOAc (pH 4.6) (Sigma) and a 2.5-fold volume of ethanol at −20°C overnight, washed with 70% ethanol and suspended in distilled H_2_O.

Prophage induction was detected by PCR with forward and reverse primers (Table S5) that were specific for each recircularized prophage and amplified across the join of prophage ends. *Se*4047 genomic DNA was used to confirm that the integrated prophage did not generate a PCR product using these primers. PCR products generated from phage particle DNA preparations were purified on QIAquick spin columns (Qiagen) and the sequences of both strands of the PCR fragments were determined using an ABI3100 DNA sequencer with BigDye fluorescent terminators and the primers used in the initial PCR amplification to confirm prophage recircularization.

### Accession numbers

The sequence and annotation of the *Se*4047 and *Sz*H70 genomes have been deposited in the EMBL database under accession numbers FM204883 and FM204884, respectively.

## Supporting Information

Table S1 Complete list of the differences between the Se4047 and SzH70 genomes. (A) Novel *S. equi* strain 4047 DNA loci. *Homologue present in *Sz*MGCS10565. (B) Novel *S. zooepidemicus* strain H70 DNA loci. *Homologue present in *Sz*MGCS10565. (C) Diversified regions of the *S. zooepidemicus* strain H70 and *S. equi* strain 4047 genomes. (D) *S. equi* strain 4047 pseudogenes (includes partial genes). (E) *S. zooepidemicus* strain H70 pseudogenes (includes partial genes).
(1.42 MB DOC)

Table S2 Sortase-processed surface proteins of *S. zooepidemicus* strain H70 and *S. equi* strain 4047. §Needleman-Wunsch global alignment; % identity given for intact CDSs only. *Orthologue present in SzMGCS10565. †Pseudogene or gene remnant. NP, not present.
(0.07 MB DOC)

Table S3 Identity of *S. equi* and *S. zooepidemicus* isolates studied and stimulation index data. NA, information not available.
(0.35 MB DOC)

Table S4 Composition of insertion elements in the *S. zooepidemicus* strain H70 and *S. equi* strain 4047 genomes. IS elements were grouped into separate isoforms (IS elements with DNA sequence more than 95% identity), numbered accordingly, and given a specific three-letter identifier to designate the species of origin. Truncated IS elements lacking either the 3-prime or 5-prime ends were not included in the table. *Chimeric IS element that appears to have been generated from recombination between ISSeq3 and ISSeq5 elements.
(0.04 MB DOC)

Table S5 Oligonucleotides used in this study.
(0.07 MB DOC)

Figure S1 Hyaluronidase treatment of Se4047, SzH70 and ST-57 (JKS115). Colony phenotypes of Se4047, SzH70 and ST-57 (JKS115) grown overnight on COBA selective agar with and without addition of hyaluronidase.
(7.65 MB TIF)

Figure S2 Alignment of SZO08560 and Sez_1114 Listeria-Bacteroides repeat domains domains. Alignment of protein domains in InlA, (Listeria monocytogenes, ABO32414), SZO08560 (SzH70), Sez_1114 (SzMGCS10565), SSU05_0473 (Streptococcus suis strain 05ZYH33, A4VTK0) and SAN_1519 (Streptococcus agalactiae strain COH1, Q3D8T2) to the Pfam hidden Markov model (HMM) for the Listeria-Bacteroides repeat domain (PF09479). Listeria-Bacteroides repeat domains are a feature of some Bacteroides forsythus proteins and families of internalins of Listeria species. Matches to the highly conserved and less well conserved Listeria-Bacteroides repeat domain residues are shown in dark and light grey respectively.
(0.70 MB TIF)
