# Supplementary material for: Genomic Evidence for the Evolution of Streptococcus equi: Host Restriction, Increased Virulence, and Genetic Exchange with Human Pathogens
Source: PLoS Pathog. 2009 Mar 27;5(3):e1000346. doi: 10.1371/journal.ppat.1000346 (PMC2654543; doi:10.1371/journal.ppat.1000346)
Supplement: Table S1 — Complete list of the differences between the Se4047 and SzH70 genomes. (A) Novel S. equi strain 4047 DNA loci. *Homologue present in SzMGCS10565. (B) Novel S. zooepidemicus strain H70 DNA loci. *Homologue present in SzMGCS10565. (C) Diversified regions of the S. zooepidemicus strain H70 and S. equi strain 4047 genomes. (D) S. equi strain 4047 pseudogenes (includes partial genes). (E) S. zooepidemicus strain H70 pseudogenes (includes partial genes). (1.42 MB DOC) [file ppat.1000346.s001.doc]

**Table S1**

**A.** Novel *S. equi* strain 4047 DNA loci.

| **No.** | **Element** | **Notes** | **CDS / region** | **Function** | **Total no. novel CDS** | **% GC** |
| --- | --- | --- | --- | --- | --- | --- |
| 1 | putative genomic island |  | SEQ0047* | putative ABC transporter ATP-binding / permease protein | 4 | 29.12 |
|  |  |  | SEQ0048* | ThiF family protein (pseudogene) |  |  |
|  |  |  | SEQ0050* | insulinase family metallopeptidase |  |  |
|  |  |  | SEQ0051* | putative regulatory protein |  |  |
| 2 |  |  | SEQ0100 | transposase (pseudogene) | 2 | 30.11 |
|  |  |  | SEQ0101* | putative exported protein |  |  |
| 3 | Prophage φSeq1 | insertion in *Se*4047 | SEQ0133 | integrase | 65 | 40.22 |
|  |  |  | SEQ0134 | hypothetical phage protein |  |  |
|  |  |  | SEQ0135 | phage repressor protein |  |  |
|  |  |  | SEQ0136 | putative phage repressor |  |  |
|  |  |  | SEQ0137 | hypothetical phage protein |  |  |
|  |  |  | SEQ0138 | hypothetical phage protein |  |  |
|  |  |  | SEQ0139 | putative DNA-binding phage protein |  |  |
|  |  |  | SEQ0140 | hypothetical phage protein |  |  |
|  |  |  | SEQ0141 | hypothetical phage protein |  |  |
|  |  |  | SEQ0142 | putative DNA-binding phage protein |  |  |
|  |  |  | SEQ0143 | hypothetical phage protein |  |  |
|  |  |  | SEQ0144 | hypothetical phage protein |  |  |
|  |  |  | SEQ0145 | hypothetical phage protein |  |  |
|  |  |  | SEQ0146 | hypothetical phage protein |  |  |
|  |  |  | SEQ0147 | hypothetical phage protein |  |  |
|  |  |  | SEQ0148 | putative phage RecT family protein |  |  |
|  |  |  | SEQ0149 | hypothetical phage protein |  |  |
|  |  |  | SEQ0150 | hypothetical phage protein (pseudogene) |  |  |
|  |  |  | SEQ0151 | hypothetical phage protein |  |  |
|  |  |  | SEQ0152 | hypothetical phage protein |  |  |
|  |  |  | SEQ0153 | hypothetical phage protein |  |  |
|  |  |  | SEQ0154 | hypothetical phage protein |  |  |
|  |  |  | SEQ0155 | hypothetical phage protein |  |  |
|  |  |  | SEQ0156 | hypothetical phage protein |  |  |
|  |  |  | SEQ0157 | putative phage membrane protein |  |  |
|  |  |  | SEQ0158 | phage DNA methylase |  |  |
|  |  |  | SEQ0159 | hypothetical phage protein |  |  |
|  |  |  | SEQ0160 | hypothetical phage protein |  |  |
|  |  |  | SEQ0161 | hypothetical phage protein |  |  |
|  |  |  | SEQ0162 | putative autolysin regulatory protein |  |  |
|  |  |  | SEQ0163 | phage DNA methylase |  |  |
|  |  |  | SEQ0164 | hypothetical phage protein |  |  |
|  |  |  | SEQ0165 | phage terminase |  |  |
|  |  |  | SEQ0166 | putative minor capsid protein |  |  |
|  |  |  | SEQ0167 | putative minor capsid protein |  |  |
|  |  |  | SEQ0168 | hypothetical phage protein |  |  |
|  |  |  | SEQ0169 | hypothetical phage protein |  |  |
|  |  |  | SEQ0170 | hypothetical phage protein |  |  |
|  |  |  | SEQ0171 | putative phage major capsid protein |  |  |
|  |  |  | SEQ0172 | hypothetical phage protein |  |  |
|  |  |  | SEQ0173 | hypothetical phage protein |  |  |
|  |  |  | SEQ0174 | hypothetical phage protein |  |  |
|  |  |  | SEQ0175 | hypothetical phage protein |  |  |
|  |  |  | SEQ0176 | hypothetical phage protein |  |  |
|  |  |  | SEQ0177 | hypothetical phage protein |  |  |
|  |  |  | SEQ0178 | hypothetical phage protein |  |  |
|  |  |  | SEQ0179 | putative phage Gp15 protein |  |  |
|  |  |  | SEQ0180 | putative phage minor tail protein |  |  |
|  |  |  | SEQ0181 | hypothetical phage protein |  |  |
|  |  |  | SEQ0182 | hypothetical phage protein |  |  |
|  |  |  | SEQ0183 | collagen-like repeat phage protein |  |  |
|  |  |  | SEQ0184 | hypothetical phage protein |  |  |
|  |  |  | SEQ0185 | hypothetical phage protein |  |  |
|  |  |  | SEQ0186 | hypothetical phage protein |  |  |
|  |  |  | SEQ0187 | hypothetical phage protein |  |  |
|  |  |  | SEQ0188 | putative phage holin |  |  |
|  |  |  | SEQ0189 | putative phage membrane protein |  |  |
|  |  |  | SEQ0190 | phage-associated cell wall hydrolase |  |  |
|  |  |  | SEQ0191 | putative DNA-binding phage protein |  |  |
|  |  |  | SEQ0192 | hypothetical phage protein |  |  |
|  |  |  | SEQ0193 | hypothetical phage protein |  |  |
|  |  |  | SEQ0194 | phage membrane protein |  |  |
|  |  |  | SEQ0195 | putative DNA-binding phage protein |  |  |
|  |  |  | SEQ0196 | hypothetical phage protein |  |  |
|  |  |  | SEQ0197 | hypothetical phage protein |  |  |
| 4 |  |  | SEQ0214 | putative DNA-binding protein (pseudogene) | 7 | 35.62 |
|  |  |  | SEQ0216* | putative membrane protein |  |  |
|  |  |  | SEQ0217* | putative membrane protein |  |  |
|  |  |  | SEQ0218* | putative membrane protein |  |  |
|  |  |  | SEQ0219* | ABC transporter, ATP-binding protein |  |  |
|  |  |  | SEQ0220* | putative membrane protein |  |  |
|  |  |  | SEQ0221* | putative membrane protein |  |  |
| 5 |  |  | SEQ0235 | factor H-binding secreted protein Se18.9 | 5 | 33.15 |
|  |  |  | SEQ0236 | putative exported protein (pseudogene) |  |  |
|  |  |  | SEQ0237 | putative membrane protein |  |  |
|  |  |  | SEQ0238 | putative membrane protein |  |  |
|  |  |  | SEQ0239 | ABC transporter, ATP-binding protein (pseudogene) |  |  |
| 6 |  |  | SEQ0281 | putative membrane protein | 1 | 30.11 |
| 7 |  |  | SEQ0307 | transposase (fragment) | 1 | 35.69 |
| 8 |  | similar region in *Sz*H70 | SEQ0309 | conserved hypothetical protein | 4 | 36.73 |
|  |  |  | SEQ0309a | putative membrane protein |  |  |
|  |  |  | SEQ0310 | hypothetical protein |  |  |
|  |  |  | SEQ0311 | hypothetical protein |  |  |
| 9 |  |  | SEQ0343 | hypothetical protein |  |  |
| 10 |  |  | SEQ0466* | fibronectin-binding protein SFS | 1 | 47.31 |
| 11 | ICE*Se1* C-terminal region |  | SEQ0753 | putative group II intron reverse transcriptase/maturase | 8 | 34.83 |
|  |  |  | SEQ0756 | putative conjugative transposon regulatory protein |  |  |
|  |  |  | SEQ0757 | putative modification methylase |  |  |
|  |  |  | SEQ0758 | putative type II restriction enzyme |  |  |
|  |  |  | SEQ0759 | putative transposase |  |  |
|  |  |  | SEQ0760 | putative transposase |  |  |
|  |  |  | SEQ0761 | conserved hypothetical protein |  |  |
|  |  |  | SEQ0762 | conserved hypothetical protein |  |  |
| 12 | Prophage φSeq2 | insertion in *Se*4047 | SEQ0787 | putative integrase | 64 | 39.24 |
|  |  |  | SEQ0788 | phage membrane protein |  |  |
|  |  |  | SEQ0789 | putative hypothetical phage protein |  |  |
|  |  |  | SEQ0790 | putative phage repressor |  |  |
|  |  |  | SEQ0791 | putative transposase |  |  |
|  |  |  | SEQ0792 | putative transposase |  |  |
|  |  |  | SEQ0793 | hypothetical phage protein |  |  |
|  |  |  | SEQ0794 | putative DNA binding phage protein |  |  |
|  |  |  | SEQ0795 | hypothetical phage protein |  |  |
|  |  |  | SEQ0796 | hypothetical phage protein |  |  |
|  |  |  | SEQ0797 | hypothetical phage protein |  |  |
|  |  |  | SEQ0798 | hypothetical phage protein |  |  |
|  |  |  | SEQ0799 | hypothetical phage protein |  |  |
|  |  |  | SEQ0800 | hypothetical phage protein |  |  |
|  |  |  | SEQ0801 | putative essential recombination function protein |  |  |
|  |  |  | SEQ0802 | putative single-strand binding protein |  |  |
|  |  |  | SEQ0803 | hypothetical phage protein |  |  |
|  |  |  | SEQ0804 | putative hypothetical phage protein |  |  |
|  |  |  | SEQ0805 | hypothetical phage protein |  |  |
|  |  |  | SEQ0806 | phage membrane protein |  |  |
|  |  |  | SEQ0807 | putative DNA methylase |  |  |
|  |  |  | SEQ0808 | putative C-5 cytosine-specific DNA methylase (pseudogene) |  |  |
|  |  |  | SEQ0810 | hypothetical phage protein |  |  |
|  |  |  | SEQ0811 | phage membrane protein |  |  |
|  |  |  | SEQ0812 | hypothetical phage protein |  |  |
|  |  |  | SEQ0813 | hypothetical phage protein |  |  |
|  |  |  | SEQ0814 | putative autolysin regulatory protein |  |  |
|  |  |  | SEQ0815 | hypothetical phage protein |  |  |
|  |  |  | SEQ0816 | hypothetical phage protein |  |  |
|  |  |  | SEQ0817 | hypothetical phage protein |  |  |
|  |  |  | SEQ0818 | putative phage HNH endonuclease |  |  |
|  |  |  | SEQ0819 | putative phage terminase, small subunit |  |  |
|  |  |  | SEQ0820 | putative phage terminase, large subunit |  |  |
|  |  |  | SEQ0821 | hypothetical phage protein |  |  |
|  |  |  | SEQ0822 | putative hypothetical phage protein |  |  |
|  |  |  | SEQ0823 | putative phage portal protein |  |  |
|  |  |  | SEQ0824 | putative phage ClpP protease |  |  |
|  |  |  | SEQ0825 | putative phage major capsid protein |  |  |
|  |  |  | SEQ0826 | hypothetical phage protein |  |  |
|  |  |  | SEQ0827 | hypothetical phage protein |  |  |
|  |  |  | SEQ0828 | hypothetical phage protein |  |  |
|  |  |  | SEQ0829 | hypothetical phage protein |  |  |
|  |  |  | SEQ0830 | hypothetical phage protein |  |  |
|  |  |  | SEQ0831 | major tail protein |  |  |
|  |  |  | SEQ0832 | hypothetical phage protein |  |  |
|  |  |  | SEQ0833 | hypothetical phage protein |  |  |
|  |  |  | SEQ0834 | putative phage tail protein |  |  |
|  |  |  | SEQ0835 | hypothetical phage protein |  |  |
|  |  |  | SEQ0836 | hypothetical phage protein |  |  |
|  |  |  | SEQ0837 | putative phage hyaluronidase |  |  |
|  |  |  | SEQ0838 | hypothetical phage protein |  |  |
|  |  |  | SEQ0839 | putative hypothetical phage protein |  |  |
|  |  |  | SEQ0840 | hypothetical phage protein |  |  |
|  |  |  | SEQ0841 | hypothetical phage protein |  |  |
|  |  |  | SEQ0842 | putative phage membrane protein |  |  |
|  |  |  | SEQ0843 | putative phage membrane protein |  |  |
|  |  |  | SEQ0844 | putative phage membrane protein |  |  |
|  |  |  | SEQ0845 | putative phage amidase protein |  |  |
|  |  |  | SEQ0846 | hypothetical phage protein |  |  |
|  |  |  | SEQ0847 | hypothetical phage membrane protein |  |  |
|  |  |  | SEQ0848 | hypothetical phage protein |  |  |
|  |  |  | SEQ0849 | phospholipase A_2_ SlaA |  |  |
|  |  |  | SEQ0850 | phage membrane protein |  |  |
|  |  |  | SEQ0851 | hypothetical phage protein |  |  |
| 13 |  |  | SEQ0902 | hypothetical protein |  |  |
| 14 |  | insertion in *Se*4047 | SEQ1101 | hypothetical protein (fragment) | 2 | 28.1 |
|  |  |  | SEQ1102 | putative conjugative transposon site-specific recombinase |  |  |
| 15 |  |  | SEQ1183 *^diverse^ | hypothetical protein | 1 | 30.26 |
| 16 | ICE*Se2* | insertion in *Se*4047 | SEQ1229 | putative conjugative transposon site-specific recombinase | 42 | 30.6 |
|  |  |  | SEQ1231 | putative DNA-binding protein |  |  |
|  |  |  | SEQ1232 | hypothetical protein |  |  |
|  |  |  | SEQ1233 | putative hydrolase EqbN |  |  |
|  |  |  | SEQ1234 | putative oxidoreductase, EqbM |  |  |
|  |  |  | SEQ1235 | ABC transporter, ATP-binding membrane protein EqbL |  |  |
|  |  |  | SEQ1236 | ABC transporter, ATP-binding membrane protein EqbK |  |  |
|  |  |  | SEQ1237 | ABC transporter, ATP-binding component EqbJ |  |  |
|  |  |  | SEQ1238 | ABC transporter permease component EqbI |  |  |
|  |  |  | SEQ1239 | ABC transporter permease component EqbH |  |  |
|  |  |  | SEQ1240 | equibactin nonribosomal peptide synthase protein EqbG |  |  |
|  |  |  | SEQ1241 | putative thiazoline reductase EqbF |  |  |
|  |  |  | SEQ1242 | equibactin nonribosomal peptide synthase protein EqbE |  |  |
|  |  |  | SEQ1243 | putative salicylate-AMP-ligase EqbD |  |  |
|  |  |  | SEQ1244 | putative 4'-phosphopantetheinyl transferase EqbC |  |  |
|  |  |  | SEQ1245 | putative non-ribosomal peptide synthesis thioesterase type II EqbB |  |  |
|  |  |  | SEQ1246 | iron-dependent repressor EqbA |  |  |
|  |  |  | SEQ1247 | putative conjugative transposon membrane protein |  |  |
|  |  |  | SEQ1249 | putative conjugative transposon mobilization protein |  |  |
|  |  |  | SEQ1250 | putative conjugative transposon mobilization protein |  |  |
|  |  |  | SEQ1251 | putative conjugative transposon membrane protein |  |  |
|  |  |  | SEQ1252 | putative conjugative transposon single-strand binding protein |  |  |
|  |  |  | SEQ1253 | putative conjugative transposon DNA recombination protein |  |  |
|  |  |  | SEQ1254 | conjugative transposon hypothetical protein |  |  |
|  |  |  | SEQ1255 | conjugative transposon hypothetical protein |  |  |
|  |  |  | SEQ1256 | putative DNA topoisomerase |  |  |
|  |  |  | SEQ1257 | putative abortive infection protein |  |  |
|  |  |  | SEQ1258 | conjugative transposon hypothetical protein |  |  |
|  |  |  | SEQ1259 | putative membrane protein |  |  |
|  |  |  | SEQ1260 | conjugative transposon hypothetical protein |  |  |
|  |  |  | SEQ1261 | putative conjugative transposon membrane protein |  |  |
|  |  |  | SEQ1262 | modification DNA methylase |  |  |
|  |  |  | SEQ1263 | putative conjugal transfer protein |  |  |
|  |  |  | SEQ1264 | putative conjugative transposon membrane protein |  |  |
|  |  |  | SEQ1265 | putative conjugative transposon membrane protein |  |  |
|  |  |  | SEQ1266 | putative conjugative transposon membrane protein |  |  |
|  |  |  | SEQ1267 | putative conjugative transposon membrane protein |  |  |
|  |  |  | SEQ1268 | putative conjugative transposon exported protein |  |  |
|  |  |  | SEQ1269 | putative ABC transporter, ATP-binding protein |  |  |
|  |  |  | SEQ1270 | putative membrane protein |  |  |
|  |  |  | SEQ1271 | putative bacteriocin |  |  |
|  |  |  | SEQ1272 | putative two-component sensor histidine kinase (fragment) |  |  |
| 17 |  |  | SEQ1273 | conserved hypothetical protein (fragment) | 3 | 34.47 |
|  |  |  | SEQ1274 | ParB-like nuclease protein |  |  |
|  |  |  | SEQ1275 | sporulation initiation inhibitor Soj homologue |  |  |
| 18 |  | deletion in *Sz*H70 and *Sz*MGCS10565 | SEQ1328 | conserved hypothetical protein (pseudogene) | 1 | 28.19 |
| 19 |  | deletion in *Sz*H70 | SEQ1517 | conserved hypothetical protein | 2 | 37.69 |
|  |  |  | SEQ1518 | hypothetical protein (fragment) |  |  |
| 20 |  |  | SEQ1654 | conserved hypothetical protein | 1 | 30.86 |
| 21 |  |  | SEQ1677a | conserved hypothetical protein (fragment) | 1 | 38.31 |
| 22 | tRNA |  | 1734575-1734638 | tRNA-Gly |  |  |
| 23 | prophage φSeq3 | insertion in *Se*4047 | SEQ1726 | hypothetical phage protein | 40 | 39.02 |
|  |  |  | SEQ1727 | exotoxin M precursor SeeM |  |  |
|  |  |  | SEQ1728 | exotoxin L precursor SeeL |  |  |
|  |  |  | SEQ1729 | putative phage amidase protein |  |  |
|  |  |  | SEQ1730 | putative phage membrane protein |  |  |
|  |  |  | SEQ1731 | putative phage membrane protein |  |  |
|  |  |  | SEQ1732 | hypothetical phage protein |  |  |
|  |  |  | SEQ1733 | hypothetical phage protein |  |  |
|  |  |  | SEQ1734 | hypothetical phage protein |  |  |
|  |  |  | SEQ1735 | hypothetical phage protein |  |  |
|  |  |  | SEQ1736 | putative collagen-like repeat protein |  |  |
|  |  |  | SEQ1737 | hypothetical phage protein |  |  |
|  |  |  | SEQ1738 | putative phage tail protein |  |  |
|  |  |  | SEQ1739 | putative phage minor tail protein |  |  |
|  |  |  | SEQ1740 | hypothetical phage protein |  |  |
|  |  |  | SEQ1741 | hypothetical phage protein |  |  |
|  |  |  | SEQ1742 | phage major tail protein |  |  |
|  |  |  | SEQ1743 | phage major tail protein |  |  |
|  |  |  | SEQ1744 | hypothetical phage protein |  |  |
|  |  |  | SEQ1745 | hypothetical phage protein |  |  |
|  |  |  | SEQ1746 | hypothetical phage protein |  |  |
|  |  |  | SEQ1747 | hypothetical phage protein |  |  |
|  |  |  | SEQ1748 | hypothetical phage protein |  |  |
|  |  |  | SEQ1749 | putative phage capsid protein |  |  |
|  |  |  | SEQ1750 | hypothetical phage protein |  |  |
|  |  |  | SEQ1751 | hypothetical phage protein |  |  |
|  |  |  | SEQ1752 | hypothetical phage protein |  |  |
|  |  |  | SEQ1753 | hypothetical phage protein |  |  |
|  |  |  | SEQ1754 | hypothetical phage protein |  |  |
|  |  |  | SEQ1755 | hypothetical phage protein |  |  |
|  |  |  | SEQ1756 | hypothetical phage protein |  |  |
|  |  |  | SEQ1757 | phage Mu protein F like protein |  |  |
|  |  |  | SEQ1758 | putative phage portal protein |  |  |
|  |  |  | SEQ1759 | putative phage terminase, large subunit |  |  |
|  |  |  | SEQ1760 | putative transposase |  |  |
|  |  |  | SEQ1761 | putative transposase |  |  |
|  |  |  | SEQ1762 | putative phage repressor protein |  |  |
|  |  |  | SEQ1763 | hypothetical phage protein |  |  |
|  |  |  | SEQ1764 | hypothetical phage protein |  |  |
|  |  |  | SEQ1765 | putative phage integrase |  |  |
| 24 |  |  | SEQ1853 | putative exported protein | 1 | 31.05 |
| 25 |  |  | SEQ1857 | putative membrane protein | 2 | 29.72 |
|  |  |  | SEQ1855a | conserved hypothetical protein |  |  |
| 26 | rRNAs / tRNAs |  | 1970594-1976601 | 16S rRNA, tRNA-Ala, 23S rRNA, 5S rRNA, tRNA-Val, tRNA-Asp, tRNA-Lys, tRNA-Leu, tRNA-Thr, tRNA-Gly, tRNA-Leu, tRNA-Arg, tRNA-Pro |  |  |
| 27 | Genomic island |  | SEQ1971 | putative plasmid stabilisation system protein | 14 | 37.18 |
|  |  |  | SEQ1972 | putative plasmid stabilisation system, antitoxin protein |  |  |
|  |  |  | SEQ1973 | conserved hypothetical protein |  |  |
|  |  |  | SEQ1974 | type I restriction-modification system R protein |  |  |
|  |  |  | SEQ1975 | anticodon nuclease |  |  |
|  |  |  | SEQ1976 | type I restriction-modification system S protein |  |  |
|  |  |  | SEQ1977 | type I restriction-modification system M protein |  |  |
|  |  |  | SEQ1978 | conserved hypothetical protein |  |  |
|  |  |  | SEQ1979 | conserved hypothetical protein |  |  |
|  |  |  | SEQ1980 | conserved hypothetical protein |  |  |
|  |  |  | SEQ1981 | conserved hypothetical protein |  |  |
|  |  |  | SEQ1982 | putative DNA-binding protein |  |  |
|  |  |  | SEQ1983 | putative DNA-binding protein |  |  |
|  |  |  | SEQ1984 | putative integrase |  |  |
| 28 |  |  | SEQ2035* | putative DNA-binding protein (fragment) | 1 | 31.28 |
| 29 | Prophage φSeq4 | insertion in *Se*4047 | SEQ2036 | exotoxin H precursor SeeH | 54 | 39.18 |
|  |  |  | SEQ2037 | exotoxin I precursor SeeI |  |  |
|  |  |  | SEQ2038 | putative phage lysin protein |  |  |
|  |  |  | SEQ2039 | putative phage membrane protein |  |  |
|  |  |  | SEQ2040 | putative phage holin |  |  |
|  |  |  | SEQ2041 | phage membrane protein |  |  |
|  |  |  | SEQ2042 | hypothetical phage protein |  |  |
|  |  |  | SEQ2043 | hypothetical phage protein |  |  |
|  |  |  | SEQ2044 | hypothetical phage protein |  |  |
|  |  |  | SEQ2045 | putative phage hyaluronidase |  |  |
|  |  |  | SEQ2046 | hypothetical phage protein |  |  |
|  |  |  | SEQ2047 | hypothetical phage protein |  |  |
|  |  |  | SEQ2048 | putative phage minor tail protein |  |  |
|  |  |  | SEQ2049 | hypothetical phage protein |  |  |
|  |  |  | SEQ2050 | hypothetical phage protein |  |  |
|  |  |  | SEQ2051 | putative phage major tail protein |  |  |
|  |  |  | SEQ2052 | hypothetical phage protein |  |  |
|  |  |  | SEQ2053 | hypothetical phage protein |  |  |
|  |  |  | SEQ2054 | hypothetical phage protein |  |  |
|  |  |  | SEQ2055 | hypothetical phage protein |  |  |
|  |  |  | SEQ2056 | hypothetical phage protein |  |  |
|  |  |  | SEQ2057 | hypothetical phage protein |  |  |
|  |  |  | SEQ2058 | hypothetical phage protein |  |  |
|  |  |  | SEQ2059 | putative phage membrane protein |  |  |
|  |  |  | SEQ2060 | putative phage minor head protein |  |  |
|  |  |  | SEQ2061 | putative phage portal protein |  |  |
|  |  |  | SEQ2062 | putative phage terminase, large subunit |  |  |
|  |  |  | SEQ2063 | putative phage terminase, small subunit |  |  |
|  |  |  | SEQ2064 | hypothetical phage protein |  |  |
|  |  |  | SEQ2065 | putative hypothetical phage protein |  |  |
|  |  |  | SEQ2066 | hypothetical phage protein |  |  |
|  |  |  | SEQ2067 | hypothetical phage protein |  |  |
|  |  |  | SEQ2068 | hypothetical phage protein |  |  |
|  |  |  | SEQ2069 | hypothetical phage protein |  |  |
|  |  |  | SEQ2070 | SNF2 family phage protein |  |  |
|  |  |  | SEQ2071 | hypothetical phage protein |  |  |
|  |  |  | SEQ2072 | putative phage DNA primase/helicase protein |  |  |
|  |  |  | SEQ2073 | putative phage DNA polymerase |  |  |
|  |  |  | SEQ2074 | hypothetical phage protein |  |  |
|  |  |  | SEQ2075 | hypothetical phage protein |  |  |
|  |  |  | SEQ2076 | hypothetical phage protein |  |  |
|  |  |  | SEQ2077 | hypothetical phage protein |  |  |
|  |  |  | SEQ2078 | hypothetical phage protein (pseudogene) |  |  |
|  |  |  | SEQ2079 | hypothetical phage protein |  |  |
|  |  |  | SEQ2080 | hypothetical phage protein |  |  |
|  |  |  | SEQ2081 | hypothetical phage protein |  |  |
|  |  |  | SEQ2082 | hypothetical phage protein |  |  |
|  |  |  | SEQ2083 | putative phage DNA-binding protein |  |  |
|  |  |  | SEQ2084 | putative phage membrane protein |  |  |
|  |  |  | SEQ2085 | putative hypothetical phage protein |  |  |
|  |  |  | SEQ2086 | putative phage DNA-binding protein |  |  |
|  |  |  | SEQ2087 | hypothetical phage protein |  |  |
|  |  |  | SEQ2088 | hypothetical phage protein |  |  |
|  |  |  | SEQ2089 | phage integrase |  |  |
| 30 |  |  | SEQ2093 | hypothetical protein (pseudogene) | 3 | 26.06 |
|  |  |  | SEQ2094* | major facilitator superfamily protein |  |  |
|  |  |  | SEQ2095 | putative exported protein |  |  |
| 31 |  | deletion in *Sz*H70 and *Sz*MGCS10565 | SEQ2170 | ABC transporter membrane protein | 4 |  |
|  |  |  | SEQ2171 | ABC transporter, ATP-binding subunit |  |  |
|  |  |  | SEQ2172 | two-component response regulator |  |  |
|  |  |  | SEQ2173 | putative two-component sensor kinase protein |  |  |

* Homologue present in *Sz*MGCS10565

**Table S1**

**B.** Novel *S. zooepidemicus* strain H70 DNA loci.

| **No.** | **Element** | **Notes** | **CDS / region** | **Function** | **Total no. novel CDS** | **% GC** |
| --- | --- | --- | --- | --- | --- | --- |
| 1 |  |  | SZO00830* | putative collagen-like cell surface-anchored protein | 1 | 54.35 |
| 2 |  |  | SZO01230 | putative exported protein | 1 | 34.02 |
| 3 |  |  | SZO01450 | putative exported protein | 1 | 28.65 |
| 4 |  | deletion in *Se*4047 | SZO01750* | sorbitol-6-phosphate 2-dehydrogenase SorD | 1 | 43.12 |
| 5 | genomic island |  | SZO02230 | integrase | 8 | 32.52 |
|  |  |  | SZO02240 | DNA-binding protein |  |  |
|  |  |  | SZO02250 | conserved hypothetical protein |  |  |
|  |  |  | SZO02260 | putative membrane protein |  |  |
|  |  |  | SZO02270 | hypothetical protein |  |  |
|  |  |  | SZO02280 | conserved hypothetical protein |  |  |
|  |  |  | SZO02290 | hypothetical protein |  |  |
|  |  |  | SZO02300 | hypothetical protein |  |  |
| 6 |  |  | SZO03350  *^diverse^ | putative exported protein | 3 | 31.92 |
|  |  |  | SZO03360 | putative exported protein |  |  |
|  |  |  | SZO03370* | putative exported protein |  |  |
| 7 | tRNAs |  | 486423 – 486623* | tRNA-Ile, tRNA-Glu |  |  |
| 8 |  | distinct restriction-modification system in *Sz*MGCS10565 | SZO04240 | putative type I restriction enzyme protein | 6 | 38.39 |
|  |  |  | SZO04250 | hypothetical protein |  |  |
|  |  |  | SZO04260 | type I restriction modification DNA specificity protein |  |  |
|  |  |  | SZO04270 | hypothetical protein |  |  |
|  |  |  | SZO04280 | putative type I restriction enzyme methylase protein |  |  |
|  |  |  | SZO04281 | hypothetical protein (fragment) |  |  |
| 9 |  |  | SZO05080 | transposase (fragment) | 8 | 30.32 |
|  |  |  | SZO05081 | conserved hypothetical protein |  |  |
|  |  |  | SZO05090 | putative permease |  |  |
|  |  |  | SZO05100 | ABC transporter protein |  |  |
|  |  |  | SZO05110 | putative lantibiotic synthetase protein |  |  |
|  |  |  | SZO05120 | prolyl oligopeptidase family protein |  |  |
|  |  |  | SZO05130 | ABC transporter protein |  |  |
|  |  |  | SZO05131 | conserved hypothetical protein (fragment) |  |  |
| 10 |  | deletion in *Se*4047 mediated by ISSeq3 elements | SZO05580 | putative lantibiotic transport ATP-binding protein | 12 | 32.45- 43.24 |
|  |  |  | SZO05590 | putative lantibiotic transport protein |  |  |
|  |  |  | SZO05600 | putative lantibiotic transport protein |  |  |
|  |  |  | SZO05610* | ABC transporter protein |  |  |
|  |  |  | SZO05620* | putative membrane protein |  |  |
|  |  |  | SZO05630* | putative membrane protein |  |  |
|  |  |  | SZO05640 | dhaKLM operon coactivator DhaQ |  |  |
|  |  |  | SZO05650 | dhaKLM operon transcriptional activator DhaS |  |  |
|  |  |  | SZO05660 | PTS-dependent dihydroxyacetone kinase, dihydroxyacetone-binding subunit DhaK |  |  |
|  |  |  | SZO05670 | PTS-dependent dihydroxyacetone kinase, ADP-binding subunit DhaL |  |  |
|  |  |  | SZO05680 | sugar phosphotransferase system (PTS), IIC component |  |  |
|  |  |  | SZO05690 | glycerol uptake facilitator protein GlpF |  |  |
| 11 |  | similar region in *Se*4047 and *Sz*MGCS10565 | SZO05820*^diverse^ | putative exported protein | 5 | 36.37 |
|  |  |  | SZO05830*^diverse^ | putative exported protein |  |  |
|  |  |  | SZO05840 | putative exported protein (pseudogene) |  |  |
|  |  |  | SZO05850 | putative exported protein |  |  |
|  |  |  | SZO05860*^diverse^ | conserved hypothetical protein |  |  |
| 12 |  | deletion in *Se*4047 and *Sz*MGCS10565 | SZO06070 | phosphate transport system protein PhoU (pseudogene) | 1 | 40.51 |
| 13 |  | deletion in *Se*4047 mediated by ISSeq3 elements | SZO06280 | hypothetical protein | 6 | 43.47 |
|  |  |  | SZO06290 *^diverse^ | putative membrane protein |  |  |
|  |  |  | SZO06300  *^diverse^ | conserved hypothetical protein |  |  |
|  |  |  | SZO06310  *^diverse^ | conserved hypothetical protein |  |  |
|  |  |  | SZO06320  *^diverse^ | putative membrane protein |  |  |
|  |  |  | SZO06330 | conserved hypothetical protein |  |  |
| 14 | genomic island |  | SZO07580 | replication initiator protein (fragment) | 12 | 31.26 |
|  |  |  | SZO07581 | conserved hypothetical protein (fragment) |  |  |
|  |  |  | SZO07590 | mobilisation protein |  |  |
|  |  |  | SZO07600 | putative relaxase (fragment) |  |  |
|  |  |  | SZO07620 | sensor histidine kinase |  |  |
|  |  |  | SZO07630 | response regulator protein |  |  |
|  |  |  | SZO07640 | putative membrane protein |  |  |
|  |  |  | SZO07660 | lantibiotic biosynthesis protein |  |  |
|  |  |  | SZO07670 | lantibiotic transport/processing ATP-binding protein |  |  |
|  |  |  | SZO07680 | putative lantibiotic ABC transporter protein |  |  |
|  |  |  | SZO07690 | putative lantibiotic ABC transporter protein |  |  |
|  |  |  | SZO07700 | putative lantibiotic ABC transporter protein |  |  |
| 15 |  | similar region in *Se*4047 and *Sz*MGCS10565 | SZO08010* | putative membrane protein | 2 | 34.11 |
|  |  |  | SZO08020* | putative membrane protein |  |  |
|  |  |  | SZO08030* | putative membrane protein |  |  |
|  |  |  | SZO08040* | putative membrane protein |  |  |
|  |  |  | SZO08050 | putative membrane protein |  |  |
| 16 |  | deletion in *Se*4047 | SZO08550* | putative recombinase | 2 | 37.64 |
|  |  |  | SZO08560* | putative cell surface-anchored protein |  |  |
| 17 | genomic island |  | SZO09570 | conserved hypothetical protein | 9 | 30.25 |
|  |  |  | SZO09580 | putative protein kinase |  |  |
|  |  |  | SZO09590 | hypothetical protein |  |  |
|  |  |  | SZO09600 | conserved hypothetical protein |  |  |
|  |  |  | SZO09610 | conserved hypothetical protein |  |  |
|  |  |  | SZO09620 | hypothetical protein |  |  |
|  |  |  | SZO09630 | type II restriction enzyme-methylase |  |  |
|  |  |  | SZO09640 | type II restriction enzyme and methylase |  |  |
|  |  |  | SZO09650 | hypothetical protein |  |  |
| 18 |  | deletion in *Se*4047 mediated by ISSeq3 elements | SZO09940* | bacteriocin-associated membrane protein | 6 | 35.92 |
|  |  |  | SZO09941* | putative bacteriocin |  |  |
|  |  |  | SZO09950* | putative thioredoxin |  |  |
|  |  |  | SZO09951* | putative bacteriocin |  |  |
|  |  |  | SZO09952* | putative bacteriocin |  |  |
|  |  |  | SZO09960* | putative membrane protein |  |  |
| 19 |  |  | SZO10340* | conserved hypothetical protein | 11 | 32.24 |
|  |  |  | SZO10361* | conserved hypothetical protein (fragment) |  |  |
|  |  |  | SZO10360* | putative exported protein |  |  |
|  |  |  | SZO10370* | conserved hypothetical protein |  |  |
|  |  |  | SZO10380* | conserved hypothetical protein |  |  |
|  |  |  | SZO10390 | conserved hypothetical protein |  |  |
|  |  |  | SZO10400 | putative exported protein |  |  |
|  |  |  | SZO10410 | conserved hypothetical protein (fragment) |  |  |
|  |  |  | SZO10420 | putative exported protein |  |  |
|  |  |  | SZO10430* | conserved hypothetical protein (pseudogene) |  |  |
|  |  |  | SZO10440* | putative membrane protein |  |  |
| 20 |  | deletion in *Se*4047 mediated by recombination between ISSeq3 and ISSeq5 elements | SZO12330* | putative ABC transporter, ATP-binding/permease protein | 8 | 33.77 |
|  |  |  | SZO12340 | putative membrane protein |  |  |
|  |  |  | SZO12350 | ABC-2 type transporter protein |  |  |
|  |  |  | SZO12360 | ABC transporter protein |  |  |
|  |  |  | SZO12370 | hypothetical protein |  |  |
|  |  |  | SZO12380 | radical SAM superfamily protein |  |  |
|  |  |  | SZO12390 | radical SAM superfamily protein |  |  |
|  |  |  | SZO12400 | putative DNA-binding protein |  |  |
| 21 | ICE*Sz1* C-terminus |  | SZO12560 | conserved hypothetical protein | 17 | 34.57 |
|  |  |  | SZO12570 | conserved hypothetical protein (fragment) |  |  |
|  |  |  | SZO12580 | conserved hypothetical protein (fragment) |  |  |
|  |  |  | SZO12590 | conserved hypothetical protein (fragment) |  |  |
|  |  |  | SZO12600 | putative DNA-binding protein |  |  |
|  |  |  | SZO12660 | DNA topoisomerase (fragment) |  |  |
|  |  |  | SZO12670 | putative replication initiation protein (fragment) |  |  |
|  |  |  | SZO12680 | putative membrane protein |  |  |
|  |  |  | SZO12690 | conserved hypothetical protein |  |  |
|  |  |  | SZO12700 | site-specific recombinase |  |  |
|  |  |  | SZO12710 | conserved hypothetical protein |  |  |
|  |  |  | SZO12720 | radical SAM superfamily protein |  |  |
|  |  |  | SZO12730 | conserved hypothetical protein |  |  |
|  |  |  | SZO12740 | conserved hypothetical protein |  |  |
|  |  |  | SZO12750 | putative DNA-binding protein |  |  |
|  |  |  | SZO12760 | conserved hypothetical protein |  |  |
|  |  |  | SZO12770 | relaxase/mobilisation protein |  |  |
| 22 | ICE*Sz1* N- terminal region |  | SZO12900 | group II intron reverse transcriptase maturase (pseudogene) | 2 | 34.23 |
|  |  |  | SZO12930 | ABC transporter protein |  |  |
| 23 |  |  | SZO13420 | putative exported protein | 1 | 34.68 |
| 24 |  |  | SZO14080* | response regulatory protein | 4 | 38.04 |
|  |  |  | SZO14090* | sensor histidine kinase |  |  |
|  |  |  | SZO14100* | ABC-2 type transporter protein |  |  |
|  |  |  | SZO14110* | ABC transporter protein |  |  |
| 25 | CRISPR locus | deletion in *Se*4047 mediated by ISSeq11 elements | 1604884 – 1606107  ^*distinct repeats^ | CRISPR repeats | 24 | 40.56 |
|  |  |  | SZO14370* | CRISPR-associated protein Cas2 |  |  |
|  |  |  | SZO14380* | CRISPR-associated protein Cas1 |  |  |
|  |  |  | SZO14390* | CRISPR-associated protein Cas4 |  |  |
|  |  |  | SZO14400* | CRISPR-associated protein |  |  |
|  |  |  | SZO14410* | CRISPR-associated protein |  |  |
|  |  |  | SZO14420* | CRISPR-associated protein |  |  |
|  |  |  | SZO14430* | CRISPR-associated helicase |  |  |
|  |  |  | SZO14440* | hypothetical protein |  |  |
|  |  |  | SZO14450* | hypothetical protein |  |  |
|  |  |  | SZO14460 | hypothetical protein |  |  |
|  |  |  | SZO14471* | conserved hypothetical protein (fragment) |  |  |
|  |  |  | SZO14470* | conserved hypothetical protein |  |  |
|  |  |  | SZO14480 | hypothetical protein |  |  |
|  |  |  | SZO14490* | conserved hypothetical protein |  |  |
|  |  |  | SZO14500* | hypothetical protein |  |  |
|  |  |  | SZO14510* | putative membrane protein |  |  |
|  |  |  | SZO14520 | conserved hypothetical protein |  |  |
|  |  |  | SZO14530 | conserved hypothetical protein |  |  |
|  |  |  | SZO14540 | hypothetical protein |  |  |
|  |  |  | SZO14550* | hypothetical protein |  |  |
|  |  |  | SZO14560 | putative membrane protein |  |  |
|  |  |  | SZO14570* | hypothetical protein |  |  |
|  |  |  | SZO14580* | hypothetical protein |  |  |
|  |  |  | SZO14590* | putative membrane protein |  |  |
| 26 | ESAT-6-like locus | deletion in *Se*4047 mediated by ISSeq3 elements | SZO14660* | ESAT-6 secretion system protein EssA | 4 | 42.29 |
|  |  |  | SZO14670* | ESAT-6 secretion system protein EsaA |  |  |
|  |  |  | SZO14680* | ESAT-6 secreted protein EsxA |  |  |
|  |  |  | SZO14690* | branched-chain amino acid transport system carrier protein |  |  |
| 27 |  |  | SZO14721* | conserved hypothetical protein (fragment) | 8 | 33.1 |
|  |  |  | SZO14730* | transport protein ComB |  |  |
|  |  |  | SZO14740* | transport/processing ATP-binding protein ComA |  |  |
|  |  |  | SZO14744* | putative competence stimulating peptide |  |  |
|  |  |  | SZO14743* | hypothetical protein |  |  |
|  |  |  | SZO14742* | putative competence stimulating peptide |  |  |
|  |  |  | SZO14741* | conserved hypothetical protein |  |  |
|  |  |  | SZO14750* | DNA-binding protein |  |  |
| 28 |  | deletion in *Se*4047 and *Sz*MGCS10565 | SZO14870 | putative exported protein | 1 | 36.51 |
| 29 |  | deletion in *Se*4047 | SZO15030* | ABC transporter, ATP-binding protein | 4 | 41.1 |
|  |  |  | SZO15040* | putative membrane protein |  |  |
|  |  |  | SZO15050* | putative membrane protein |  |  |
|  |  |  | SZO15060* | conserved hypothetical protein |  |  |
| 30 |  | deletion in *Se*4047 | SZO15110* | D-ribose-binding protein precursor RbsD | 6 | 43.95 |
|  |  |  | SZO15120* | ribose transport system permease protein RbsC |  |  |
|  |  |  | SZO15130* | ribose import ATP-binding protein RbsA |  |  |
|  |  |  | SZO15140* | high affinity ribose transport protein RbsD |  |  |
|  |  |  | SZO15150* | ribokinase RbsK |  |  |
|  |  |  | SZO15160* | ribose operon repressor RbsR |  |  |
| 31 |  | deletion in *Se*4047 | SZO15220* | 6-phospho-beta-galactosidase LacG | 5 | 44.11 |
|  |  |  | SZO15230* | sugar phosphotransferase system (PTS), lactose-specific IICB component LacE |  |  |
|  |  |  | SZO15240* | sugar phosphotransferase system (PTS), lactose-specific phosphotransferase enzyme IIA component LacF |  |  |
|  |  |  | SZO15250* | transcription antiterminator LacT |  |  |
|  |  |  | SZO15260* | tagatose 1,6-diphosphate aldolase LacD |  |  |
| 32 |  | similar region in *Se*4047 | SZO17200 | hypothetical protein | 12 | 37.97 |
|  |  |  | SZO17210 | hypothetical protein |  |  |
|  |  |  | SZO17220 | hypothetical protein |  |  |
|  |  |  | SZO17230 | hypothetical protein |  |  |
|  |  |  | SZO17240 | hypothetical protein |  |  |
|  |  |  | SZO17250 | hypothetical protein |  |  |
|  |  |  | SZO17260 | conserved hypothetical protein |  |  |
|  |  |  | SZO17270 | hypothetical protein |  |  |
|  |  |  | SZO17280 | hypothetical protein |  |  |
|  |  |  | SZO17290 | conserved hypothetical protein (fragment) |  |  |
|  |  |  | SZO17300* | conserved hypothetical protein |  |  |
|  |  |  | SZO17310 | conserved hypothetical protein |  |  |
| 33 |  |  | SZO17550 | conserved hypothetical protein | 2 | 29.63 |
|  |  |  | SZO17560 | hypothetical protein |  |  |
| 34 | ICE*Sz2* | insertion in *Sz*H70 | SZO17561 | relaxase (fragment) | 41 | 38.64 |
|  |  |  | SZO17570 | group II intron-encoded protein (pseudogene) |  |  |
|  |  |  | SZO17580 | putative relaxase (fragment) |  |  |
|  |  |  | SZO17590 | mobilisation protein |  |  |
|  |  |  | SZO17600 | conserved hypothetical protein |  |  |
|  |  |  | SZO17610 | DNA-binding protein |  |  |
|  |  |  | SZO17620 | conserved hypothetical protein |  |  |
|  |  |  | SZO17630 | conserved hypothetical protein |  |  |
|  |  |  | SZO17640 | conserved hypothetical protein |  |  |
|  |  |  | SZO17650 | conserved hypothetical protein |  |  |
|  |  |  | SZO17660 | conserved hypothetical protein (pseudogene) |  |  |
|  |  |  | SZO17680 | putative exported protein |  |  |
|  |  |  | SZO17690 | multi antimicrobial extrusion (MATE) family transporter |  |  |
|  |  |  | SZO17700 | conserved hypothetical protein |  |  |
|  |  |  | SZO17710 | radical SAM superfamily protein |  |  |
|  |  |  | SZO17730 | transcriptional regulator |  |  |
|  |  |  | SZO17740 | putative membrane protein |  |  |
|  |  |  | SZO17750 | conserved hypothetical protein |  |  |
|  |  |  | SZO17760 | putative helicase |  |  |
|  |  |  | SZO17770 | putative group II intron reverse transcriptase/maturase |  |  |
|  |  |  | SZO17790 | conserved hypothetical protein |  |  |
|  |  |  | SZO17800 | putative glucan-binding protein |  |  |
|  |  |  | SZO17810 | hypothetical protein |  |  |
|  |  |  | SZO17820 | conserved hypothetical protein |  |  |
|  |  |  | SZO17830 | conserved hypothetical protein |  |  |
|  |  |  | SZO17840 | putative amidase (fragment) |  |  |
|  |  |  | SZO17850 | putative peptidase (fragment) |  |  |
|  |  |  | SZO17860 | conserved hypothetical protein (pseudogene) |  |  |
|  |  |  | SZO17870 | putative group II intron reverse transcriptase/maturase |  |  |
|  |  |  | SZO17890 | conserved hypothetical protein (pseudogene) |  |  |
|  |  |  | SZO17900 | putative membrane protein |  |  |
|  |  |  | SZO17910 | putative membrane protein |  |  |
|  |  |  | SZO17920 | TraG family protein (pseudogene) |  |  |
|  |  |  | SZO17940 | conserved hypothetical protein |  |  |
|  |  |  | SZO17950 | abortive infection protein |  |  |
|  |  |  | SZO17960 | conserved hypothetical protein |  |  |
|  |  |  | SZO17970 | conserved hypothetical protein (fragment) |  |  |
|  |  |  | SZO17980 | conserved hypothetical protein (fragment) |  |  |
|  |  |  | SZO17990 | C-5 cytosine-specific DNA methylase |  |  |
|  |  |  | SZO18000 | replication initiator A protein |  |  |
|  |  |  | SZO18010 | conserved hypothetical protein |  |  |
| 35 |  | deletion in *Se*4047 mediated by ISSeq3, 2 distinct pilus clusters in *Sz*MGCS10565 | SZO18270 | sortase SrtC.2 | 8 | 37.91 |
|  |  |  | SZO18280 | sortase SrtC.3 |  |  |
|  |  |  | SZO18290 | sortase SrtC.4 |  |  |
|  |  |  | SZO18300 | putative exported protein |  |  |
|  |  |  | SZO18310 | putative ancillary pilus subunit |  |  |
|  |  |  | SZO18320 | putative backbone pilus subunit |  |  |
|  |  |  | SZO18330 | putative pilus subunit |  |  |
|  |  |  | SZO18340* | AraC family regulatory protein |  |  |
| 36 | genomic island | insertion in *Sz*H70 and *Sz*MGCS10565 into the end of *rpsD* | SZO19050* | plasmid stabilization system protein | 4 | 36.88 |
|  |  |  | SZO19060* | conserved hypothetical protein |  |  |
|  |  |  | SZO19061* | phage hypothetical protein (pseudogene) |  |  |
|  |  |  | SZO19062* | phage hypothetical protein (fragment) |  |  |
| 37 |  | similar region in *Se*4047 | SZO19300* | putative membrane protein | 1 | 34.61 |
|  |  |  | SZO19310* | putative membrane protein |  |  |
|  |  |  | SZO19320* | putative membrane protein |  |  |
|  |  |  | SZO19330 | putative membrane protein |  |  |

* Homologue present in *Sz*MGCS10565

**Table S1**

**C.** Diversified regions of the *S. zooepidemicus* strain H70 and *S. equi* strain 4047 genomes. § Needleman-Wunsch global alignment of amino acid sequences. † Pseudogene or partial gene. Regions 1 to 33 are also diverse in *Sz*MGCS10565 relative to *Se*4047 and/or *Sz*H70 (a homologous CDS is absent in *Sz*MGCS10565 in regions 7, 23 and 30).

| **No.** | ***Se*4047** | ***Sz*H70** | **% Similarity** § | **Function** |
| --- | --- | --- | --- | --- |
| 1 | SEQ0084 | SZO00790 | 78.9 | hypothetical protein |
| 2 | SEQ0090 | SZO00840 | 66.7 | putative collagen-like cell surface-anchored protein SclG |
| 3 | SEQ0121 | SZO01080 | 77.0 | putative membrane protein |
| 4 | SEQ0202 | SZO01211† | 77.8 | putative exported protein |
|  | SEQ0203 | SZO01220 | 88.8 | putative exported protein |
| 5 | SEQ0256 | SZO01590 | 81.2 | putative cell surface-anchored protein |
|  | SEQ0260 | SZO01630 | 84.7 | putative collagen-like cell surface-anchored protein SclH |
| 6 | SEQ0280 | SZO17540 | 76.5 | putative collagen-like cell surface-anchored protein SclD |
| 7 | SEQ0308 | SZO17320 | 71.6 | membrane protein |
| 8 | SEQ0367 | SZO16670 | 76.2 | MerR family regulatory protein |
| 9 | SEQ0375† | SZO16630 | 71.5 | collagen- and fibronectin-binding protein Fne/Fnz |
| 10 | SEQ0402 | SZO16370 | 71.9 | putative cell surface-anchored protein |
| 11 | SEQ0432 | SZO16140 | 90.7 | endoglycosidase EndoS |
| 12 | SEQ0521 | SZO15350 | 91.7 | putative negative regulator of copper transport operon CopY |
| 13 | SEQ0527 | SZO15290 | 93.6 | galactose-6-phosphate isomerase LacA subunit |
| 14 | SEQ0555 | SZO14890 | 45.1 | putative collagen and fibronectin-binding cell surface-anchored protein FneE |
| 15 | SEQ0566† | SZO14790 | 59.3 | Szp/SzPSe-like cell surface-anchored protein |
| 16 | SEQ0633 | SZO13850 | 80.1 | putative collagen-like surface-anchored protein SclE |
| 17 | SEQ0855 | SZO12230 | 83.3 | putative collagen-like surface-anchored protein SclF |
| 18 | SEQ0896 | SZO11861 | 63.4 | putative exported protein |
| 19 | SEQ0933 | SZO11530 | 84.2 | fibrinogen-binding cell surface-anchored protein SzPSe; adhesin and hypervariable protective antigen Szp |
| 20 | SEQ0938 | SZO11480 | 81.3 | Mac family IgG endopeptidase antiphagocytic and neutrophil-binding protein IdeE/IdeZ |
|  | SEQ0939 | SZO11470 | 75.5 | putative cell surface-anchored protein |
| 21 | SEQ1051 | SZO10440 | 51.0 | putative membrane protein |
| 22 | SEQ1510 | SZO06420 | 82.2 | conserved hypothetical protein |
|  | SEQ1511 | SZO06410† | 88.5 | hypothetical protein |
| 23 | SEQ1543 | SZO06080 | 85.2 | putative exported protein |
| 24 | SEQ1564 | SZO05860 | 58.7 | conserved hypothetical protein |
|  | SEQ1565 | SZO05820 | 73.5 | putative membrane protein |
| 25 | SEQ1606 | SZO05380 | 67.3 | putative collagen-binding collagen-like surface-anchored protein FneC |
| 26 | SEQ1607† | SZO05350 | 48.3 | putative collagen-binding surface-anchored protein FneD |
| 27 | SEQ1649 | SZO04910 | 63.1 | putative collagen-like surface-anchored protein FneF |
| 28 | SEQ1670 | SZO04720 | 44.9 | putative exported protein |
| 29 | SEQ1817 | SZO03720 | 79.3 | collagen-like cell surface-anchored protein SclI |
| 30 | SEQ1941 | SZO02570 | 74.6 | putative membrane protein |
| 31 | SEQ1999 | SZO02080 | 85.0 | fibronectin and collagen-binding cell surface anchored protein FneB/Fnz2 |
| 32 | SEQ2017 | SZO01900 | 74.7 | antiphagocytic cell surface-anchored fibrinogen- and IgG Fc-binding protein |
| 33 | SEQ2101 | SZO18110 | 69.1 | putative collagen-like surface-anchored protein SclC |

**Table S1**

**D.** *S. equi* strain 4047 pseudogenes (includes partial genes). † indicates partial gene

| **No.** | ***Se*4047** | ***Sz*H70 ortholog** | **Intact in *Sz*H70** | **Function** |
| --- | --- | --- | --- | --- |
| 1 | SEQ0036† | SZO00360 | no | conserved hypothetical protein |
| 2 | SEQ0036a† | SZO00370 | no | conserved hypothetical protein |
| 3 | SEQ0038 | SZO00390 | yes | putative DNA-binding protein |
| 4 | SEQ0048 | not present | - | ThiF family protein |
| 5 | SEQ0083 | SZO00780 | no | transposase |
| 6 | SEQ0085 | SZO00800 | yes | putative membrane protein |
| 7 | SEQ0100† | not present | - | putative transposase |
| 8 | SEQ0115 | not present | - | transposase |
| 9 | SEQ0150 (φSeq1) | not present | - | hypothetical phage protein |
| 10 | SEQ0214 | not present | - | putative DNA-binding protein |
| 11 | SEQ0232† | SZO01430 | yes | putative cell wall anchored protein Spa |
| 12 | SEQ0236 | not present | - | putative exported protein |
| 13 | SEQ0239 | not present | - | ABC transporter, ATP-binding protein |
| 14 | SEQ0273† | SZO17520 | yes | putative rhomboid family membrane protein |
| 15 | SEQ0275 | not present | - | transposase |
| 16 | SEQ0275 | not present | - | transposase |
| 17 | SEQ0307† | not present | - | transposase |
| 18 | SEQ0309a† | not present | - | putative membrane protein |
| 19 | SEQ0313 | SZO17180 | yes | hypothetical protein |
| 20 | SEQ0320 | SZO17120 | yes | putative N-acetylmannosamine-6-phosphate 2-epimerase |
| 21 | SEQ0328† | SZO17040 | yes | putative acetyl xylan esterase (AXE1) family protein |
| 22 | SEQ0370 | SZO16650 | yes | response regulator SaeR |
| 23 | SEQ0375 | SZ016630 | yes | Fne collagen and fibronectin-binding protein |
| 24 | SEQ0380 | SZO16601 | yes | putative DNA-binding protein |
| 25 | SEQ0405† | SZO16340 | no | putative hypothetical phage protein |
| 26 | SEQ0424 | SZO16200 | yes | conserved hypothetical protein |
| 27 | SEQ0426 | SZO16190 | yes | conserved hypothetical protein |
| 28 | SEQ0438 | not present | - | transposase |
| 29 | SEQ0443 | SZO16070 | yes | Shr heme binding cell surface protein |
| 30 | SEQ0495 | SZO15570 | yes | putative haloacid dehalogenase-like hydrolase |
| 31 | SEQ0509a† | SZO15450 | no | putative integrase |
| 32 | SEQ0509b† | SZO15440 | no | putative transposase |
| 33 | SEQ0511† | SZO15430 | no | putative membrane protein |
| 34 | SEQ0514† | SZO15420 | no | conserved hypothetical protein |
| 35 | SEQ0530† | SZO15260 | yes | putative tagatose 1,6-diphosphate aldolase |
| 36 | SEQ0531† | SZO15220 | yes | 6-phospho-beta-galactosidase LacG |
| 37 | SEQ0541† | SZO15030 | yes | ABC transporter, ATP-binding protein |
| 38 | SEQ0556† | not present | - | Scl surface protein |
| 39 | SEQ0566 | SZO14790 | yes | M-like surface-anchored protein |
| 40 | SEQ0573† | not present | - | transposase |
| 41 | SEQ0576† | SZO14660 | yes | ESAT-6 secretion system protein EssA |
| 42 | SEQ0578 | SZO14651 | yes | ESAT-6 secretion system protein EsaB |
| 43 | SEQ0583a† | SZO14590 | yes | putative membrane protein |
| 44 | SEQ0584† | SZO14360 | yes | transposase |
| 45 | SEQ0585† | SZO14350 | yes | transposase |
| 46 | SEQ0663 | SZO13550 | yes | transposase |
| 47 | SEQ0685 | SZO13370 | yes | putative competence protein CoiA-like family protein |
| 48 | SEQ0738 | SZO12890 | yes | putative membrane protein on ICESe1 |
| 49 | SEQ0733 | SZO12940 | no | putative conjugal transfer protein |
| 50 | SEQ0746† | SZO12820 | yes | putative DNA topoisomerase on ICESe1 |
| 51 | SEQ0749† | SZO12660 | yes | putative DNA topoisomerase on ICESe1 |
| 52 | SEQ0763† | SZO12770 | yes | putative relaxase/mobilization protein end of ICESe1 but not end of ICEsz1 |
| 53 | SEQ0774 | SZO12430 | yes | putative oligopeptidase |
| 54 | SEQ0780† | SZO12330 | yes | putative ABC transporter, ATP-binding/permease protein |
| 55 | SEQ0808 (φSeq2) | not present | - | putative C-5 cytosine-specific DNA methylase |
| 56 | SEQ0866 | SZO12130 | yes | putative beta-galactosidase precursor |
| 57 | SEQ0871 | SZO12110 | yes | sugar phosphotransferase system (PTS), sorbose-specific family, IIC component |
| 58 | SEQ0899† | SZO11830 | no | transposase |
| 59 | SEQ0909 | SZO11740 | yes | putative 1,4-alpha-glucan branching enzyme |
| 60 | SEQ0934 | SZO11520 | yes | TetR family regulatory protein |
| 61 | SEQ0943† | SZO11420 | no | transposase |
| 62 | SEQ0986 | not present | - | transposase |
| 63 | SEQ1007 | not present | - | transposase |
| 64 | SEQ1064 | SZO10210 | yes | putative ABC transporter permease protein |
| 65 | SEQ1068 | not present | - | transposase |
| 66 | SEQ1077 | SZO10150 | yes | putative cell surface-anchored C5A peptidase precursor |
| 67 | SEQ1088 | SZO10050 | yes | conserved hypothetical protein |
| 68 | SEQ1098† | SZO09960 | yes | putative membrane protein |
| 69 | SEQ1101† | not present | - | hypothetical protein |
| 70 | SEQ1103† | SZO08820 | yes | putative ABC transporter ATP-binding membrane protein |
| 71 | SEQ1149 | SZO09270 | yes | putative ABC-type glycine betaine transport system ATP-binding protein |
| 72 | SEQ1151 | SZO09280 | yes | putative glycine betaine ABC transporter permease and substrate binding protein |
| 73 | SEQ1155 | SZO09310 | yes | ApbE family protein |
| 74 | SEQ1189 | SZO09690 | yes | putative ABC transporter permease |
| 75 | SEQ1209 | SZO09840 | yes | putative ammonia monooxygenase |
| 76 | SEQ1223 | SZO09930 | yes | putative ABC transporter ATP-binding protein |
| 77 | SEQ1225† | SZO09940 | yes | bacteriocin-associated membrane protein |
| 78 | SEQ1226† | SZO09950 | yes | putative thioredoxin (bacteriocin cluster) |
| 79 | SEQ1272† | not present | - | putative two-component sensor histidine kinase (on start of ICESe2) |
| 80 | SEQ1273† | not present | - | hypothetical protein (upstream of ICESe2) |
| 81 | SEQ1276† | SZO08820 | yes | putative ABC transporter ATP-binding membrane protein |
| 82 | SEQ1281 | SZO08770 | yes | putative ABC transporter, ATP-binding/permease protein |
| 83 | SEQ1284 | SZO08750 | yes | putative ABC transporter ATP-binding protein |
| 84 | SEQ1307a† | SZO08560 | yes | putative cell surface-anchored protein |
| 85 | SEQ1328 | not present | - | conserved hypothetical protein |
| 86 | SEQ1364† | SZO07970 | no | putative uncharacterized protein |
| 87 | SEQ1380† | SZO07810 | no | putative membrane protein (bacteriocin associated) |
| 88 | SEQ1383† | SZO07780 | no | putative bacteriocin transport/processing ATP-binding protein |
| 89 | SEQ1385 | SZO07770 | no | putative bacteriocin secretion protein |
| 90 | SEQ1387 | SZO07750 | no | putative transposase |
| 91 | SEQ1467 | SZO06800 | yes | aminopeptidase PepS |
| 92 | SEQ1479 | SZO06680 | yes | hyaluronate lyase precursor HysA |
| 93 | SEQ1492 | SZO06560 | yes | conserved hypothetical protein |
| 94 | SEQ1494 | SZO06550 | yes | conserved hypothetical protein |
| 95 | SEQ1507 | SZO06440 | yes | putative membrane (in operon with RNA polymerase sigma factor protein) |
| 96 | SEQ1518† | SZO06361 | no | hypothetical protein |
| 97 | SEQ1523 | SZO06260 | yes | major facilitator superfamily protein |
| 98 | SEQ1528† | SZO06220 | no | transposase |
| 99 | SEQ1573 | not present | - | putative transposase |
| 100 | SEQ1585† | SZO05580 | yes | putative lantibiotic transport ATP-binding protein |
| 101 | SEQ1588a† | SZO05541 | no | conserved hypothetical protein |
| 102 | SEQ1589† | SZO05540 | no | putative transposase |
| 103 | SEQ1590† | SZO05530 | no | putative transposase |
| 104 | SEQ1607 | SZO05350 | yes | collagen-binding surface-anchored protein |
| 105 | SEQ1613 | SZO05300 | yes | sugar phosphotransferase system (PTS), IIC component |
| 106 | SEQ1632† | SZO05140 | no | conserved hypothetical protein |
| 107 | SEQ1667 | SZO04750 | yes | conserved hypothetical protein |
| 108 | SEQ1671 | SZO04710 | yes | putative exported protein |
| 109 | SEQ1677† | SZO04650 | no | conserved hypothetical protein |
| 110 | SEQ1677a† | not present | - | conserved hypothetical protein |
| 111 | SEQ1699 | SZO04441 | no | putative exported protein |
| 112 | SEQ1720† | SZO04200 | no | transposase |
| 113 | SEQ1724 | SZO04180 | yes | putative late competence protein ComFC |
| 114 | SEQ1725 | SZO04170 | yes | putative late competence protein ComFA |
| 115 | SEQ1810 | SZO03760 | yes | putative exported protein |
| 116 | SEQ1824a† | SZO03651 | no | transposase |
| 117 | SEQ1854† | SZO03390 | yes | conserved hypothetical protein |
| 118 | SEQ1855a† | not present | - | conserved hypothetical protein |
| 119 | SEQ1873 | not present | - | transposase |
| 120 | SEQ1924 | not present | - | transposase |
| 121 | SEQ1942† | SZO02560 | no | integrase |
| 122 | SEQ1966† | SZO02330 | no | transposase |
| 123 | SEQ1968† | SZO02310 | no | transposase |
| 124 | SEQ2010 | SZO01970 | yes | conserved hypothetical protein |
| 125 | SEQ2035† | not present | - | putative DNA-binding protein |
| 126 | SEQ2078  (φSeq4) | not present | - | hypothetical phage protein |
| 127 | SEQ2093 | not present | - | hypothetical protein |
| 128 | SEQ2099 | SZO18092 | yes | conserved hypothetical protein |
| 129 | SEQ2117 | not present | - | transposase |
| 130 | SEQ2119† | SZO18340 | yes | AraC family regulatory protein |
| 131 | SEQ2122 | SZO18370 | yes | deoxynucleoside kinase |
| 132 | SEQ2138 | SZO18520 | yes | putative membrane protein |
| 133 | SEQ2184 | SZO18940 | yes | conserved hypothetical protein |
| 134 | SEQ2192 | SZO18990 | yes | putative MutT/NUDIX hydrolase family protein |
| 135 | SEQ2225 | not present | - | transposase |
| 136 | SEQ2227 | SZO19320 | yes | putative membrane protein |

**Table S1**

**E.** *S. zooepidemicus* strain H70 pseudogenes (includes partial genes). † indicates partial gene

| **No.** | ***Sz*H70** | ***Se*4047 ortholog** | **Intact in *Se4047*** | **Function** |
| --- | --- | --- | --- | --- |
| 1 | SZO00360† | SEQ0036 | no | conserved hypothetical protein |
| 2 | SZO00370† | SEQ0036a | no | conserved hypothetical protein |
| 3 | SZO00780† | SEQ0083 | no | transposase |
| 4 | SZO01211 | SEQ0202 | yes | putative exported protein |
| 5 | SZO01310 | SEQ0213 | yes | putative mutator protein MutX |
| 6 | SZO01811† | SEQ2027 | yes | NUDIX hydrolase |
| 7 | SZO01890 | SEQ2018 | yes | AraC family regulatory protein |
| 8 | SZO02310† | SEQ1968 | no | transposase |
| 9 | SZO02330† | SEQ1966 | no | transposase |
| 10 | SZO02560† | SEQ1942 | no | integrase |
| 11 | SZO03651† | SEQ1824a | no | transposase |
| 12 | SZO04200† | SEQ1720 | no | transposase |
| 13 | SZO04281† | not present | - | hypothetical protein |
| 14 | SZO04441 | SEQ1699 | No | putative exported protein |
| 15 | SZO04650† | SEQ1677 | no | conserved hypothetical protein |
| 16 | SZO05080† | not present | - | transposase |
| 17 | SZO05131† | not present | - | conserved hypothetical protein |
| 18 | SZO05140† | SEQ1632 | no | conserved hypothetical protein |
| 19 | SZO05460 | SEQ1598 | yes | putative ABC transporter, ATP-binding/permease protein |
| 20 | SZO05530† | SEQ1590 | no | putative transposase |
| 21 | SZO05540† | SEQ1589 | no | putative transposase |
| 22 | SZO05541† | SEQ1588a | no | transposase |
| 23 | SZO05550 | SEQ1588 | yes | putative lantibiotic leader peptide processing serine protease |
| 24 | SZO05840 | not present | - | putative exported protein |
| 25 | SZO06070 | not present | - | phosphate transport system protein PhoU |
| 26 | SZO06220† | SEQ1528 | no | transposase |
| 27 | SZO06320† | not present | - | putative membrane protein |
| 28 | SZO06360† | SEQ1516 | yes | hypothetical protein |
| 29 | SZO06361† | SEQ1518 | no | hypothetical protein |
| 30 | SZO06380 | SEQ1514 | yes | hypothetical protein |
| 31 | SZO06410† | SEQ1511 | yes | hypothetical protein |
| 32 | SZO07580† | not present | - | replication initiator protein (on genomic island) |
| 33 | SZO07581† | not present | - | conserved hypothetical protein |
| 34 | SZO07600† | not present | - | putative relaxase |
| 35 | SZO07660 | not present | - | lantibiotic biosynthesis protein |
| 36 | SZO07710† | SEQ1389 | yes | transposase |
| 37 | SZO07750 | SEQ1387 | no | putative transposase |
| 38 | SZO07770 | SEQ1385 | no | putative bacteriocin secretion protein |
| 39 | SZO07780† | SEQ1383 | no | putative bacteriocin transport/processing ATP-binding protein |
| 40 | SZO07790 | SEQ1382 | yes | putative bacteriocin |
| 41 | SZO07810† | SEQ1380 | no | putative membrane protein (bacteriocin associated) |
| 42 | SZO07970† | SEQ1364 | no | putative uncharacterized protein |
| 43 | SZO08000 | SEQ1361 | yes | putative membrane protein |
| 44 | SZO10201† | SEQ1066 | yes | transposase |
| 45 | SZO10361† | not present | - | conserved hypothetical protein |
| 46 | SZO10410† | not present | - | conserved hypothetical protein |
| 47 | SZO10430 | not present | - | conserved hypothetical protein |
| 48 | SZO11420† | SEQ0943 | no | transposase |
| 49 | SZO11830† | SEQ0899 | no | transposase |
| 50 | SZO12460 | SEQ0771 | yes | putative membrane protein |
| 51 | SZO12570† | not present | - | conserved hypothetical protein |
| 52 | SZO12580† | not present | - | conserved hypothetical protein |
| 53 | SZO12590† | not present | - | conserved hypothetical protein |
| 54 | SZO12620† | SEQ0752 | no | putative conjugative transposon DNA recombination protein |
| 55 | SZO12630† | SEQ0752 | no | putative conjugative transposon DNA recombination protein |
| 56 | SZO12640 | SEQ0751 | yes | DNA-binding protein |
| 57 | SZO12660† | not present | - | DNA topoisomerase |
| 58 | SZO12670† | not present | - | putative replication initiation protein |
| 59 | SZO12900 | not present | - | group II intron reverse transcriptase maturase |
| 60 | SZO12921 | SEQ0737 | yes | Putative conjugative transposon membrane protein |
| 61 | SZO12940 | SEQ0733 | no | putative conjugal transfer protein |
| 62 | SZO14260 | SEQ0595 | yes | arginine repressor |
| 63 | SZO14360 | SEQ0584 | no | putative transposase |
| 64 | SZO14471† | not present | - | conserved hypothetical protein |
| 65 | SZO14721† | not present | - | conserved hypothetical protein |
| 66 | SZO15430† | SEQ0511 | no | putative membrane protein |
| 67 | SZO15420† | SEQ0514 | no | conserved hypothetical protein |
| 68 | SZO15440† | SEQ0509b | no | putative transposase |
| 69 | SZO15450† | SEQ0509a | no | putative integrase |
| 70 | SZO16340† | SEQ0405 | no | putative hypothetical phage protein |
| 71 | SZO16870 | SEQ0346 | yes | acid phosphatase precursor LppC |
| 72 | SZO17290† | SEQ0308 | yes | conserved hypothetical protein |
| 73 | SZO17330† | not present | - | transposase |
| 74 | SZO17561† | not present | - | relaxase |
| 75 | SZO17570 | not present | - | group II intron-encoded protein |
| 76 | SZO17580† | not present | - | putative relaxase |
| 77 | SZO17660 | not present | - | conserved hypothetical protein |
| 78 | SZO17840† | not present | - | putative amidase |
| 79 | SZO17850† | not present | - | putative peptidase |
| 80 | SZO17860 | not present | - | conserved hypothetical protein |
| 81 | SZO17890 | not present | - | conserved hypothetical protein |
| 82 | SZO17920 | not present | - | TraG family protein |
| 83 | SZO17970† | not present | - | conserved hypothetical protein |
| 84 | SZO17980† | not present | - | conserved hypothetical protein |
| 85 | SZO18020† | SEQ1389 | yes | putative transposase |
| 86 | SZO18030† | SEQ1389 | yes | putative transposase |
| 87 | SZO18031† and SZO01811† | SEQ2027 | yes | NUDIX hydrolase |
| 88 | SZO18820† | SEQ2170 | yes | ABC transporter membrane protein |
| 89 | SZO18970† | SEQ2190 | yes | putative cell surface-anchored protein |
| 90 | SZO19061 | not present | - | phage hypothetical protein |
| 91 | SZO19062† | not present | - | phage hypothetical protein |
