# Supplementary material for: Genomic Evidence for the Evolution of Streptococcus equi: Host Restriction, Increased Virulence, and Genetic Exchange with Human Pathogens
Source: PLoS Pathog. 2009 Mar 27;5(3):e1000346. doi: 10.1371/journal.ppat.1000346 (PMC2654543; doi:10.1371/journal.ppat.1000346)
Supplement: Table S2 — Sortase-processed surface proteins of S. zooepidemicus strain H70 and S. equi strain 4047. §Needleman-Wunsch global alignment; % identity given for intact CDSs only. *Orthologue present in SzMGCS10565. †Pseudogene or gene remnant. NP, not present. (0.07 MB DOC) [file ppat.1000346.s002.doc]

**Table S2.** Sortase-processed surface proteins of *S. zooepidemicus* strain H70 and *S. equi* strain 4047. § Needleman-Wunsch global alignment; % identity given for intact CDSs only. * Orthologue present in *Sz*MGCS10565. NP Not present. † Pseudogene or gene remnant

| ***Se*4047** | ***Sz*H70** | **% identity§** | **Product (putative sortase motif)** |
| --- | --- | --- | --- |
| NP | SZO00830* | - | putative collagen-like surface-anchored protein (LPATG) |
| SEQ0090* | SZO00840* | 52.6 | putative collagen-like surface-anchored protein SclG (LPATG) |
| SEQ0232*† | SZO01430* | - | putative cell surface-anchored protein (LPSTG) |
| SEQ0256* | SZO01590* | 74.3 | putative cell surface-anchored protein (LPATA) |
| SEQ0260* | SZO01630* | 82.2 | putative collagen-like cell surface-anchored protein SclH (LPATG) |
| SEQ0280* | SZO17540* | 76.5 | putative collagen-like cell surface-anchored protein SclD (LPATG) |
| SEQ0375†* | SZO16630* | 65.4 | fibronectin-binding protein Fne/Fnz (LPQTN) |
| SEQ0402* | SZO16370* | 61.6 | putative cell surface-anchored protein (LPSTG) |
| SEQ0555* | SZO14890* | 39.0 | putative collagen and fibronectin-binding cell surface-anchored protein FneE (LPRTN) (SZO14890 has a collagen-like domain) |
| SEQ0556†* | NP | - | putative collagen-like surface-anchored protein (LPKTN)* |
| SEQ0563* | SZO14810* | 97.8 | chemokine protease ScpC (LPSTG) |
| SEQ0566†* | SZO14790* | - | SzP/SzPSe-like cell surface-anchored protein (LPSTG) |
| SEQ0633* | SZO13850* | 75.0 | putative collagen-like surface-anchored protein SclE (LPATG) |
| SEQ0646* | SZO13730* | 95.5 | putative cell surface-anchored protein (LPLTG) |
| SEQ0721* | SZO13070* | 99.5 | Ig, α_2_-macroglobulin and albumin binding protein Eag/Zag (LPTTG) |
| SEQ0855* | SZO12230* | 77.0 | putative collagen-like cell surface-anchored protein SclF (LPSTG) |
| SEQ0904* | SZO11790* | 97.4 | endonuclease/exonuclease/phosphatase family surface anchored protein (LPKTG) |
| SEQ0933* | SZO11530* | 79.3 | SzPSe: fibrinogen-binding cell surface-anchored protein; SzP: adhesin and hypervariable protective antigen (LPSTG) |
| SEQ0935* | SZO11510* | 88.9 | collagen binding, putative ancillary pilus subunit Cne (LPDTG) |
| SEQ0936* | SZO11500* | 98.3 | putative backbone pilus subunit (T6-antigen-like) (LPSTG) |
| SEQ0939* | SZO11470* | 66.7 | putative cell surface-anchored protein (LPSTG) |
| SEQ0944* | SZO11410* | 98.2 | putative cell surface-anchored pullulanase (LPKTG) |
| SEQ1077†* | SZO10150* | - | putative cell surface-anchored C5A peptidase (LPKTS) |
| SEQ1116* | SZO08940* | 93.2 | putative cell surface-anchored protein (LPKTG) |
| SEQ1226†* | SZO09950* | - | putative cell surface-anchored thioredoxin (FPKTG) |
| SEQ1278* | SZO08800* | 99.4 | putative cell surface-anchored 5'-nucleotidase (LPATG) |
| SEQ1307a†* | SZO08560* | - | putative cell surfaced-anchored protein (LPKTG) |
| SEQ1606 | SZO05380 | 56.4 | putative collagen-binding collagen-like cell surface-anchored protein FneC (LPKTN) |
| SEQ1607†* | SZO05350* | - | putative collagen-binding surface-anchored protein FneD (LPKTN) |
| SEQ1649* | SZO04910* | 59.7 | putative collagen-binding collagen-like surface-anchored protein FneF (LPKTN) |
| SEQ1817* | SZO03720* | 74.2 | collagen-like surface-anchored protein SclI (LPATG) |
| SEQ1959* | SZO02400* | 95.1 | putative cell surface-anchored protein (LPRSG) |
| SEQ1999* | SZO02080* | 76.5 | fibronectin-binding protein FneB/Fnz2 (LPKTH) |
| SEQ2017* | SZO01900* | 65.8 | antiphagocytic cell surface-anchored fibrinogen- and IgG Fc-binding protein SeM/SzM (LPSTG) |
| SEQ2100* | SZO18100* | 98.7 | putative cell surface-anchored protein (LPATG) |
| SEQ2101* | SZO18110* | 58.8 | putative collagen-like surface-anchored protein SclC (LPATG) |
| NP | SZO18310 | - | putative ancillary pilus subunit (VPYTG) |
| NP | SZO18320 | - | putative backbone pilus subunit (IPQTG) |
| NP | SZO18330 | - | putative pilus subunit (FPMTG) |
| SEQ2180* | SZO18890* | 75.3 | putative cell surface-anchored protein (LPATG) |
| SEQ2190* | SZO18970†* | - | putative cell surface-anchored protein (LPATG) |
