# Supplementary material for: Genomic Evidence for the Evolution of Streptococcus equi: Host Restriction, Increased Virulence, and Genetic Exchange with Human Pathogens
Source: PLoS Pathog. 2009 Mar 27;5(3):e1000346. doi: 10.1371/journal.ppat.1000346 (PMC2654543; doi:10.1371/journal.ppat.1000346)
Supplement: Table S3 — Identity of S. equi and S. zooepidemicus isolates studied and stimulation index data. NA, information not available. (0.35 MB DOC) [file ppat.1000346.s003.doc]

**Table S3.** Identity of *S. equi* and *S. zooepidemicus* isolates studied and stimulation index data.

| **Species** | **ST** | **Strain ID** | **Disease/ Source** | **Animal** | **Year** | **Stimulation index** |
| --- | --- | --- | --- | --- | --- | --- |
| *Sz* | 19 | B27 3726.1 | Uterine infection/abortion | Horse | 2007 | 1.5 |
| *Sz* | 101 | 3604 | Uterine infection/abortion | Horse | 2006 | 1.2 |
| *Sz* | 101 | B24 2382 | Uterine infection/abortion | Horse | 2004 | 1.6 |
| *Sz* | 101 | B24 4996 | Uterine infection/abortion | Horse | 2004 | 1.7 |
| *Sz* | 142 | 3727 | Uterine infection/abortion | Horse | 2007 | 1.1 |
| *Sz* | 56 | B23 3322 | Uterine infection/abortion | Horse | 2003 | 1.7 |
| *Se* | 179 | 3154 | Strangles | Horse | 2004 | 2.8 |
| *Se* | 179 | 8229 | Strangles | Dog | 2004 | 3.3 |
| *Se* | 179 | B23 7325 | Strangles | Horse | 2003 | 2.5 |
| *Se* | 179 | 4047 | Strangles | Horse | 1990 | 4.2 |
| *Se* | 179 | SA | Strangles | Horse | 1999 | 2.9 |
| *Se* | 179 | 303 | Strangles | Horse | 1999 | 4 |
| *Se* | 179 | 7060 | Strangles | Horse | 2003 | 2.9 |
| *Se* | 179 | 1931 | Strangles | Horse | 2004 | 4.3 |
| *Se* | 179 | 1351 | Strangles | Horse | 2004 | 3.1 |
| *Se* | 179 | 1350 | Strangles | Horse | 2003 | 3.3 |
| *Se* | 179 | 7344 | Strangles | Horse | 2003 | 2.8 |
| *Se* | 179 | 7140 | Strangles | Horse | 2003 | 2.8 |
| *Se* | 179 | 7171 | Strangles | Horse | 2003 | 3.5 |
| *Se* | 179 | 7364 | Strangles | Horse | 2003 | 3.1 |
| *Se* | 179 | 7326 | Strangles | Horse | 2003 | 2.3 |
| *Se* | 179 | 3155 | Strangles | Horse | 2004 | 3.6 |
| *Se* | 179 | 3156 | Strangles | Horse | 2004 | 3.2 |
| *Se* | 179 | 181063 | Strangles | Horse | 1999 | 3 |
| *Se* | 179 | JKS 044 | Strangles carrier | Horse | 2006 | 2.6 |
| *Se* | 179 | JKS 043 | Strangles | Horse | 2006 | 3.4 |
| *Se* | 179 | JKS 063 | Strangles | Horse | 2006 | 3 |
| *Se* | 179 | JKS 225 | Strangles | Horse | 2006 | 3.2 |
| *Se* | 179 | CF32 | Strangles | Horse | 1981 | 2.6 |
| *Se* | 179 | JKS 559 | Strangles | Horse | 2006 | 2.2 |
| *Se* | 151 | 7329 | Strangles | Horse | 2006 | 2.7 |
| *Se* | 151 | Mo | Strangles brain abscess | Horse | 2008 | 2.7 |
| *Sz* | 49 | B25 4861.1 | Wound infection | Horse | 2005 | 0.9 |
| *Sz* | 49 | B23 6378 | Uterine infection/abortion | Horse | 2003 | 0.8 |
| *Sz* | 49 | B27 1175.2 | Uterine infection/abortion | Horse | 2007 | 1.1 |
| *Sz* | 49 | B26 8276 | Upper respiratory | Horse | 2006 | 1.3 |
| *Sz* | 137 | 628 | Uterine infection/abortion | Horse | 2007 | 1.0 |
| *Sz* | 149 | 6619 | Uterine infection/abortion | Horse | 2003 | 1.4 |
| *Sz* | 6 | S13 | Skin swab | Dog | 2002 | 1.7 |
| *Sz* | 133 | 2258 | Foot swab | Horse | 2007 | 1.6 |
| *Sz* | 10 | BHS32 | Lower respiratory | Dog | 2001 | 1.0 |
| *Sz* | 10 | BHS41 | Lower respiratory | Dog | 2001 | 1 |
| *Sz* | 10 | B26 6443 | Wound infection | Horse | 2006 | 1.3 |
| *Sz* | 22 | BHS53 | Lower respiratory | Dog | 2001 | 1.1 |
| *Sz* | 47 | BHS 28 | Lower respiratory | Dog | 2001 | 1.2 |
| *Sz* | 55 | B23 7163 | Uterine infection/abortion | Horse | 2003 | 2.1 |
| *Sz* | 15 | B23 6907 | Uterine infection/abortion | Horse | 2003 | 1.1 |
| *Sz* | 15 | B27 2806.2 | Uterine infection/abortion | Horse | 2007 | 1.0 |
| *Sz* | 91 | BHS 37 | Lower respiratory | Dog | 2001 | 1.1 |
| *Sz* | 110 | 4847 | Upper respiratory | Horse | 1996 | 1.5 |
| *Sz* | 119 | 4904 | Upper respiratory | Horse | 1996 | 1.1 |
| *Sz* | 119 | 5382 | Upper respiratory | Horse | 1996 | 1.0 |
| *Sz* | 45 | 605851 | Upper respiratory | Horse | 1996 | 1.2 |
| *Sz* | 45 | 5845 | Upper respiratory | Horse | 1996 | 1.5 |
| *Sz* | 45 | D56 | Lower respiratory | Horse | 2000 | 0.7 |
| *Sz* | 147 | 6875 | Uterine infection/abortion | Horse | 2006 | 1.4 |
| *Sz* | 51 | B23 6074 | Uterine infection/abortion | Horse | 2003 | 1.7 |
| *Sz* | 143 | 3512 | Uterine infection/abortion | Horse | 2007 | 1.3 |
| *Sz* | 128 | 8718.2 | Uterine infection/abortion | Horse | 2006 | 1.9 |
| *Sz* | 111 | 5831 | Upper respiratory | Horse | 1996 | 1.8 |
| *Sz* | 126 | 5079 | Uterine infection/abortion | Horse | 2006 | 2.4 |
| *Sz* | 131 | 3543 | Foot swab | Horse | 2006 | 0.9 |
| *Sz* | 16 | B24 7132 | Upper respiratory | Horse | 2004 | 1.1 |
| *Sz* | 140 | B27 3530.1 | Wound infection | Horse | 2007 | 1.3 |
| *Sz* | 134 | 2567.1 | Udder swab | Horse | 2007 | 1.4 |
| *Sz* | 57 | JKS 115 | Upper respiratory | Horse | 2006 | 2.4 |
| *Sz* | 1 | H70 | Upper respiratory | Horse | 2000 | 1.4 |
| *Sz* | 1 | B27 7271 | Uterine infection/abortion | Horse | 2003 | 1.5 |
| *Sz* | 1 | B27 0479 | Uterine infection/abortion | Horse | 2007 | 1.3 |
| *Sz* | 71 | 60 5632 | Upper respiratory | Horse | 1996 | 1.0 |
| *Sz* | 71 | 60 5013 | Upper respiratory | Horse | 1996 | 1.3 |
| *Sz* | 104 | 8278 | Upper respiratory | Horse | 2006 | 1.2 |
| *Sz* | 104 | 8275 | Upper respiratory | Horse | 2006 | 1.2 |
| *Sz* | 108 | 5938 | Upper respiratory | Horse | 1996 | 1.3 |
| *Sz* | 108 | 4853 | Upper respiratory | Horse | 1996 | 0.9 |
| *Sz* | 123 | BHS5 | Lower respiratory | Dog | 2001 | 6.7 |
| *Sz* | 127 | 6360 | Wound infection | Horse | 2006 | 5.0 |
| *Sz* | 141 | 482 | Uterine infection/abortion | Horse | 2007 | 5.8 |
| *Sz* | 7 | B26 8900 | Wound infection | Horse | 2006 | 5.5 |
| *Sz* | 7 | B27 1185 | Uterine infection/abortion | Horse | 2007 | 3.5 |
| *Sz* | 7 | 60 2333 | Upper respiratory | Horse | 1996 | 4.9 |
| *Sz* | 48 | B25 4433 | Lymph node abscess | Horse | 2005 | 3.3 |
| *Sz* | 70 | B26 8310 | Upper respiratory | Horse | 2006 | 3.3 |
| *Sz* | 70 | B26 8277 | Upper respiratory | Horse | 2006 | 2.6 |
| *Sz* | 5 | B24 7159 | Lymph node abscess | Horse | 2004 | 5.4 |
| *Sz* | 5 | B24 7043 | Lymph node abscess | Horse | 2004 | 2.9 |
| *Sz* | 53 | B24 7102.2 | Lymph node abscess | Horse | 2004 | 7.1 |
| *Sz* | 8 | H8 | Lower respiratory | Horse | 2000 | 9.6 |
| *Sz* | 8 | D40 | Lower respiratory | Horse | 2000 | 5.3 |
| *Sz* | 8 | D2a | Lower respiratory | Horse | 2000 | 5.1 |
| *Sz* | 46 | B25 0061 | Lymph node abscess | Horse | 2005 | 3.6 |
| *Sz* | 113 | 4885 | Upper respiratory | Horse | 1996 | 6.5 |
| *Sz* | 113 | 5617 | Upper respiratory | Horse | 1996 | 5.3 |
| *Sz* | 96 | B25 4763 | Lymph node abscess | Horse | 2005 | 1.0 |
| *Sz* | 20 | B25 4042.1 | NA | Horse | 2005 | 1.7 |
| *Sz* | 118 | 3050 | Nasal fluid | Horse | 2007 | 1.5 |
| *Sz* | 118 | 4901 | Upper respiratory | Horse | 1996 | 1.4 |
| *Sz* | 118 | 2329 | Upper respiratory | Horse | 1996 | 2.0 |
| *Sz* | 139 | 972395 | Upper respiratory | Horse | 1997 | 0.8 |
| *Sz* | 146 | 8250 | Wound infection | Horse | 2006 | 1.1 |
| *Sz* | 58 | B26 Tansey | Nephritis | Horse | 2006 | 1.3 |
| *Sz* | 61 | B26 8269.1 | Wound infection | Horse | 2006 | 1.0 |
| *Sz* | 61 | B26 0225 | Uterine infection/abortion | Horse | 2006 | 1.1 |
| *Sz* | 94 | B23 6170 | Uterine infection/abortion | Horse | 2003 | 1.7 |
| *Sz* | 26 | B27 2596 | Uterine infection/abortion | Horse | 2007 | 1.3 |
| *Sz* | 26 | B26 6993 | Uterine infection/abortion | Horse | 2006 | 1.6 |
| *Sz* | 124 | 5808 | Wound infection | Horse | 2003 | 0.7 |
| *Sz* | 135 | 2265 | Intra-abdomen pus | Horse | 2007 | 1.8 |
| *Sz* | 39 | B26 6334 | Wound infection | Horse | 2006 | 1.0 |
| *Sz* | 39 | JKS 241 | Upper respiratory | Horse | 2007 | 1.3 |
| *Sz* | 106 | 605849 | Upper respiratory | Horse | 1996 | 8.0 |
| *Sz* | 106 | 5936 | Upper respiratory | Horse | 1996 | 3.2 |
| *Sz* | 106 | 8306 | Upper respiratory | Horse | 2006 | 1.5 |
| *Sz* | 107 | 8307 | Upper respiratory | Horse | 2006 | 0.7 |
| *Sz* | 9 | H54 | Upper respiratory | Horse | 2000 | 1.9 |
| *Sz* | 120 | 4897 | Upper respiratory | Horse | 1996 | 3.5 |
| *Sz* | 63 | B26 2253 | Uterine infection/abortion | Horse | 2006 | 1.4 |
| *Sz* | 63 | B26 3540 | Foot swab | Horse | 2006 | 1.2 |
| *Sz* | 54 | B24 7155 | Upper respiratory | Horse | 2004 | 1.6 |
| *Sz* | 130 | 7101 | Wound infection | Horse | 2006 | 1.6 |
| *Sz* | 50 | B25 5405 | Lymph node abscess | Horse | 2005 | 1.5 |
| *Sz* | 138 | 917 | Uterine infection/abortion | Horse | 2007 | 1.0 |
| *Sz* | 82 | B26 6458 | Uterine infection/abortion | Horse | 2006 | 1.6 |
| *Sz* | 178 | 2958 | Uterine infection/abortion | Horse | 2007 | 1.6 |
| *Sz* | 109 | 5820 | Upper respiratory | Horse | 1996 | 1.3 |
| *Sz* | 150 | 8311 | Upper respiratory | Horse | 2006 | 1.0 |
| *Sz* | 150 | 8308 | Upper respiratory | Horse | 2006 | 1.8 |
| *Sz* | 4 | B27 4388 | Uterine infection/abortion | Horse | 2007 | 1.3 |
| *Sz* | 4 | B24 4389 | Uterine infection/abortion | Horse | 2004 | 1.4 |
| *Sz* | 132 | 2853 | Lymph node abscess | Horse | 2007 | 0.8 |
| *Sz* | 132 | 1913 | Uterine infection/abortion | Horse | 2007 | 1.0 |
| *Sz* | 103 | 8300 | Upper respiratory | Horse | 2006 | 1.0 |
| *Sz* | 103 | 8297 | Upper respiratory | Horse | 2006 | 1.2 |
| *Sz* | 116 | 8293 | Upper respiratory | Horse | 1996 | 1.1 |
| *Sz* | 112 | 4866 | Upper respiratory | Horse | 1996 | 1.0 |
| *Sz* | 112 | 5951 | Upper respiratory | Horse | 1996 | 1.2 |
| *Sz* | 122 | 4871 | Upper respiratory | Horse | 1996 | 1.4 |
| *Sz* | 93 | B23 7166 | Uterine infection/abortion | Horse | 2003 | 1.0 |
| *Sz* | 121 | 5185 | Upper respiratory | Horse | 2006 | 1.9 |
| *Sz* | 3 | D33 | Lower respiratory | Horse | 2000 | 1.8 |
| *Sz* | 92 | B23 4314B | NA | Horse | 2003 | 2.0 |
| *Sz* | 125 | 7157 | Upper respiratory | Horse | 2004 | 1.6 |
| *Sz* | 136 | 3101 | Uterine infection/abortion | Horse | 2007 | 0.8 |
| *Sz* | 97 | B26 5081.1 | Uterine infection/abortion | Horse | 2006 | 1.0 |
| *Sz* | 97 | B26 8570 | Uterine infection/abortion | Horse | 2006 | 1.2 |
| *Sz* | 97 | B26 8571 | Uterine infection/abortion | Horse | 2006 | 1.6 |
| *Sz* | 97 | B27 2742.2 | Uterine infection/abortion | Horse | 2007 | 1.1 |
| *Sz* | 97 | B27 7714 | Lymph node abscess | Horse | 2007 | 1.5 |
| *Sz* | 97 | B27 0624.2 | Uterine infection/abortion | Horse | 2007 | 1.1 |
| *Sz* | 97 | B27 0624.1 | Uterine infection/abortion | Horse | 2007 | 1.6 |
| *Sz* | 97 | B26 8575 | Uterine infection/abortion | Horse | 2006 | 1.4 |
| *Sz* | 99 | B27 0739 | Uterine infection/abortion | Horse | 2007 | 1.4 |
| *Sz* | 117 | 5623 | Lower respiratory | Horse | 1996 | 1.8 |
| *Sz* | 148 | 4875 | Lower respiratory | Horse | 1996 | 1.3 |
| *Sz* | 2 | D14a | Lower respiratory | Horse | 2000 | 2.4 |
| *Sz* | 12 | B25 0590 | Wound infection | Horse | 2005 | 0.9 |
| *Sz* | 13 | B26 0863 | Uterine infection/abortion | Horse | 2006 | 1.7 |
| *Sz* | 18 | 1770 | Fatal haemorrhagic pneumoniae | Dog | 2008 | 0.9 |
| *Sz* | 18 | 1727 | Fatal haemorrhagic pneumoniae | Dog | 2008 | 1.0 |
| *Sz* | 95 | B24 7156 | Upper respiratory | Horse | 2004 | 1.1 |
| *Sz* | 98 | B26 8337 | Keratitis | Horse | 2006 | 1.1 |
| *Sz* | 98 | B27 2247 | Uterine infection/abortion | Horse | 2007 | 0.8 |
| *Sz* | 100 | B26 3593 | Keratitis | Horse | 2006 | 1.5 |
| *Sz* | 102 | 8299 | Upper respiratory | Horse | 2006 | 1.8 |
| *Sz* | 115 | 4893 | Upper respiratory | Horse | 1996 | 2.0 |
| *Sz* | 144 | 2410 | Upper respiratory | Horse | 1997 | 3.0 |

NA Information not available
