# Supplementary material for: Genomic Evidence for the Evolution of Streptococcus equi: Host Restriction, Increased Virulence, and Genetic Exchange with Human Pathogens
Source: PLoS Pathog. 2009 Mar 27;5(3):e1000346. doi: 10.1371/journal.ppat.1000346 (PMC2654543; doi:10.1371/journal.ppat.1000346)
Supplement: Table S4 — Composition of insertion elements in the S. zooepidemicus strain H70 and S. equi strain 4047 genomes. IS elements were grouped into separate isoforms (IS elements with DNA sequence more than 95% identity), numbered accordingly, and given a specific three-letter identifier to designate the species of origin. Truncated IS elements lacking either the 3-prime or 5-prime ends were not included in the table. *Chimeric IS element that appears to have been generated from recombination between ISSeq3 and ISSeq5 elements. (0.04 MB DOC) [file ppat.1000346.s004.doc]

**Table S4.** Composition of insertion elements in the *S. zooepidemicus* strain H70 and *S. equi* strain 4047 genomes. IS elements were grouped into separate isoforms (IS elements with DNA sequence more than 95% identity), numbered accordingly, and given a specific 3 letter identifier to designate the species of origin. Truncated IS elements lacking either the 3-prime or 5-prime ends were not included in the table. *chimeric IS element that appears to have been generated from recombination between ISSeq3 and ISSeq5 elements.

| **Insertion element** | ***Sz*H70** | ***Se*4047** | **Family** |
| --- | --- | --- | --- |
| ISSzo1 (ISSeq1) | 4 | 4 | ISL3 |
| ISSzo2 (ISSeq2) | 5 | 3 | ISNCY |
| ISSzo3 (ISSeq3) | 4 | 40 | IS3 |
| ISSzo4 (ISSeq4) | 5 | 11 | IS1634 |
| ISSzo5 (ISSeq5) | 2 | 8 | IS3 |
| ISSzo6 | 1 | 0 | IS30 |
| ISSzo7 | 3 | 0 | IS3 |
| ISSzo8 (ISSeq8) | 2 | 2 | IS200/IS605 |
| ISSzo9 (ISSeq9) | 1 | 1 | IS200/IS605 |
| ISSzo10 | 2 | 0 | ISAs1 |
| ISSzo11 (ISSeq11) | 1 | 1 | ISL3 |
| ISSeq14 | 0 | 1 | ISL3 |
| ISSeq15* | 0 | 1 | IS3 |
| **Total** | 30 | 73 |  |
