# Supplementary material for: Genomic Evidence for the Evolution of Streptococcus equi: Host Restriction, Increased Virulence, and Genetic Exchange with Human Pathogens
Source: PLoS Pathog. 2009 Mar 27;5(3):e1000346. doi: 10.1371/journal.ppat.1000346 (PMC2654543; doi:10.1371/journal.ppat.1000346)
Supplement: Table S5 — Oligonucleotides used in this study. (0.07 MB DOC) [file ppat.1000346.s005.doc]

**Table S5.** Oligonucleotides used in this study.

| **Primer** | **Sequence (5’-3’)** | **Gene / region** |
| --- | --- | --- |
| zm457 | GGGTTAATGAGCCGATACTCTTTG | *lacE* (SZO15230) |
| zm458 | CGTTCCTAATACCAAGCCAAGC | *lacE* (SZO15230) |
| 18f | CGATGTAGCTAAGTTGGCTG | *rbsD* (SZO15140) |
| 18r | AGGTGTATCCTCTCCTGTTC | *rbsD* (SZO15140) |
| 28f | CGGGTAAGACAGTGATTGTC | *sorD* (SZO01750) |
| 28r | ACCAAAATGCGCCCAACAGC | *sorD* (SZO01750) |
| 66f | AACGCTTGTGGCTTTCTCC | *hysA* (SZO06680) |
| 66r | ACTCTTTGCCCCTCTTGATC | *hysA* (SZO06680) |
| zm49 | GACGAATTCAGAGTATGCGCGAATGCTAG | *srtC.2* - *srtC.3* (SZO18270 - SZO18280) |
| zm50 | GACGATATCACCAAGCATCATCAAAAGGTAG | *srtC.2* - *srtC.3* (SZO18270 - SZO18280) |
| 24f | GTATTTGGAACAGTAGCACCG | SZO08560 |
| 24r | CATCCTTAACATGCTTCGCC | SZO08560 |
| zm492 | CAGCAACCTTTCATTACCAAATTACC | *esaA* |
| zm493 | AACAAAGACTTCAAATCAATCACAGC | *esaA* |
| zm432 | GTGTGTTGATTATGGCCAGC | Conserved hypothetical protein CRISPR locus (SZO14370) |
| zm433 | TATCAGAACGCCCTAGTGTC | Conserved hypothetical protein CRISPR locus (SZO14370) |
| 1f | CAGATGATGTTCTAGAGATGGG | *slaA* (SEQ0849) |
| 1r | CTCTAATAGCATCGGCTACG | *slaA* (SEQ0849) |
| zm381 | CAAGTGCAACTATGGCAACAG | *slaB* (SEQ2155) |
| zm382 | ATCCTGCCTTGAAATACTTTCG | *slaB* (SEQ2155) |
| zm455 | TTAATACGGATGAAAATACTACAGTTTGG | *seeL* (SEQ1728) |
| zm456 | CAACATTAAACATTCTTTCCTGTGAAG | *seeL* (SEQ1728) |
| 4f | CTGTTAGGATGGTTTCTGCG | *seeM* (SEQ1727) |
| 4r | TCAGCCGATAATGCAAGACC | *seeM* (SEQ1727) |
| 15f | CAAGAGGCTTGTGAATGTCC | *seeH* (SEQ2036) |
| 15r | CATGCTATTAAAGTCTCCATTGCC | *seeH* (SEQ2036) |
| 15af | TTGGAGTATTCTCCTCCCTG | *seeI* (SEQ2037) |
| 15ar | AGCATACTCTCTCTGTCACC | *seeI* (SEQ2037) |
| *eqbE* f | AAGATATAGCAGCATCGTATCG | *eqbE* (SEQ1242) |
| *eqbE* r | TCTAAATCTCTATTAAATAGCGGTATATTG | *eqbE* (SEQ1242) |
| zm340 | ACTGATATTGAAGAAGCTTGAAG | *se18.9* (SEQ0235) |
| zm341 | GCATAGGTGTCAGTCAAAGC | *se18.9* (SEQ0235) |
| *gyrA* f | AAGGCGGGATTCCTAAAATC | *gyrA* (SEQ1170, SZO09430) |
| *gyrA* r | GATAAGTAAGCCCTCTAAAATGTG | *gyrA* (SEQ1170, SZO09430) |
| Phiseq1F | GATAAGGAGGTGTTTATGC | across junction of circular φseq1 |
| Phiseq1R | CCATATCTCAGTTGCTTG | across junction of circular φseq1 |
| Phiseq2F | GGAGGTTGTGACAGACG | across junction of circular φseq2 |
| Phiseq2R | GCAGCACTAGGTCATTCTAC | across junction of circular φseq2 |
| Phiseq3F | GTGATAACGATGATGAACTTG | across junction of circular φseq3 |
| Phiseq3R | GCAAGCTATTGATCATGG | across junction of circular φseq3 |
| Phiseq4F | GACAATCGAGCGGATATC | across junction of circular φseq4 |
| Phiseq4R | GTTGGACATGCTGACTCG | across junction of circular φseq4 |
| zm235 | ATTGGGAACACCTTGCAAGG | across junction of circular ICE*Se2* |
| zm236 | TTTTTTCTTCTTCCCACTGGC | across junction of circular ICE*Se2* |
| zm473F | TTAGTTTCTTTGCTTGTATTTGGAACAG | SZO08560 |
| zm474R | TCTGCGTGTTTACTTCATCAGTATTATC | SZO08560 |
| zm475R | CTTTTCTCTCATATATCCCTTTTCTTG | Recombinase (SZO08550) |
| zm476F | AGGAGCAAAGCTTCTTGATG | Recombinase (SZO08550) |
