# Supplementary figures and images for: Genomic Evidence for the Evolution of Streptococcus equi: Host Restriction, Increased Virulence, and Genetic Exchange with Human Pathogens
Source: PLoS Pathog. 2009 Mar 27;5(3):e1000346. doi: 10.1371/journal.ppat.1000346 (PMC2654543; doi:10.1371/journal.ppat.1000346)

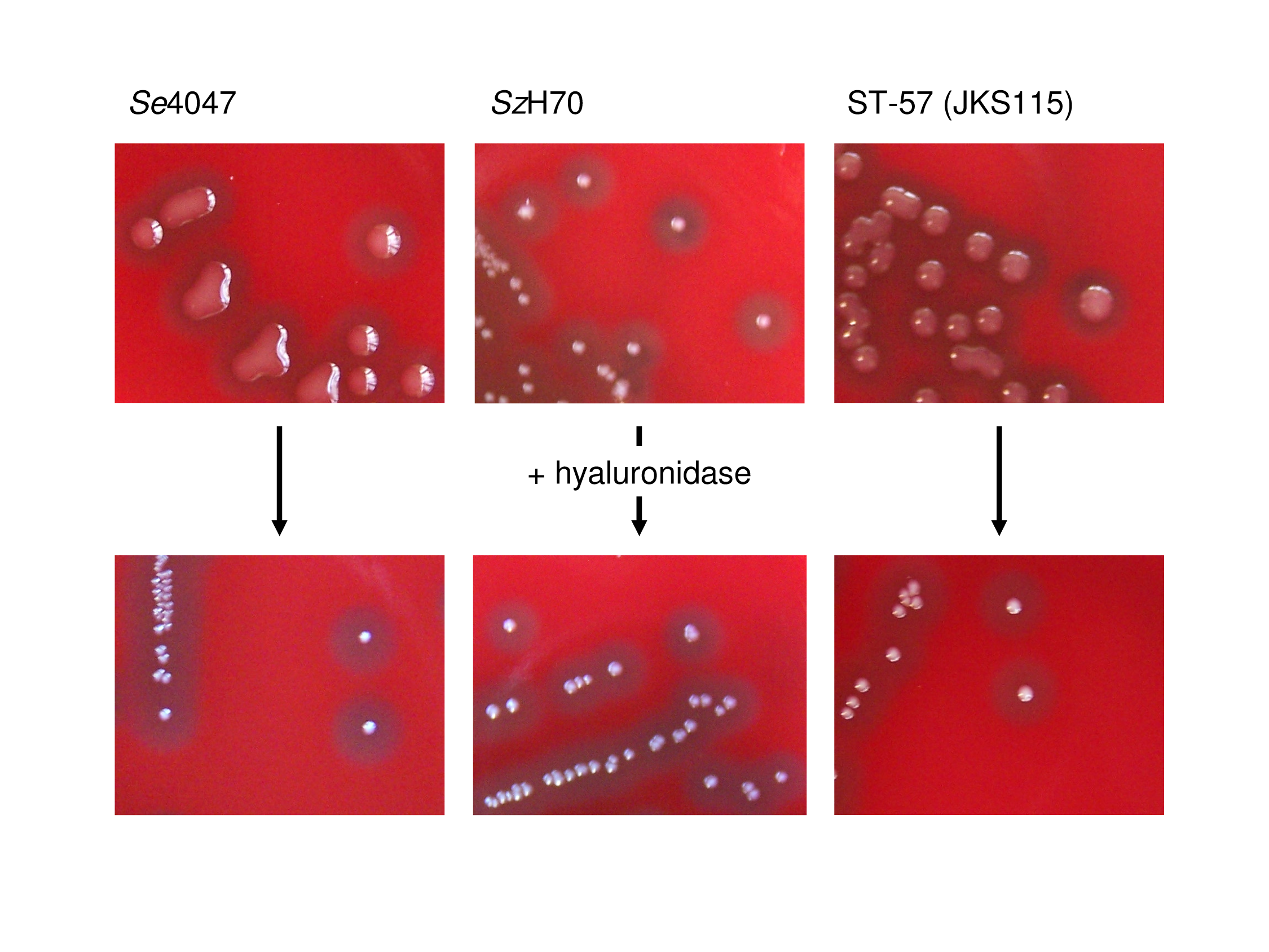

Supplement: Figure S1 — Hyaluronidase treatment of Se4047, SzH70 and ST-57 (JKS115). Colony phenotypes of Se4047, SzH70 and ST-57 (JKS115) grown overnight on COBA selective agar with and without addition of hyaluronidase. (7.65 MB TIF) [file ppat.1000346.s006.tif]

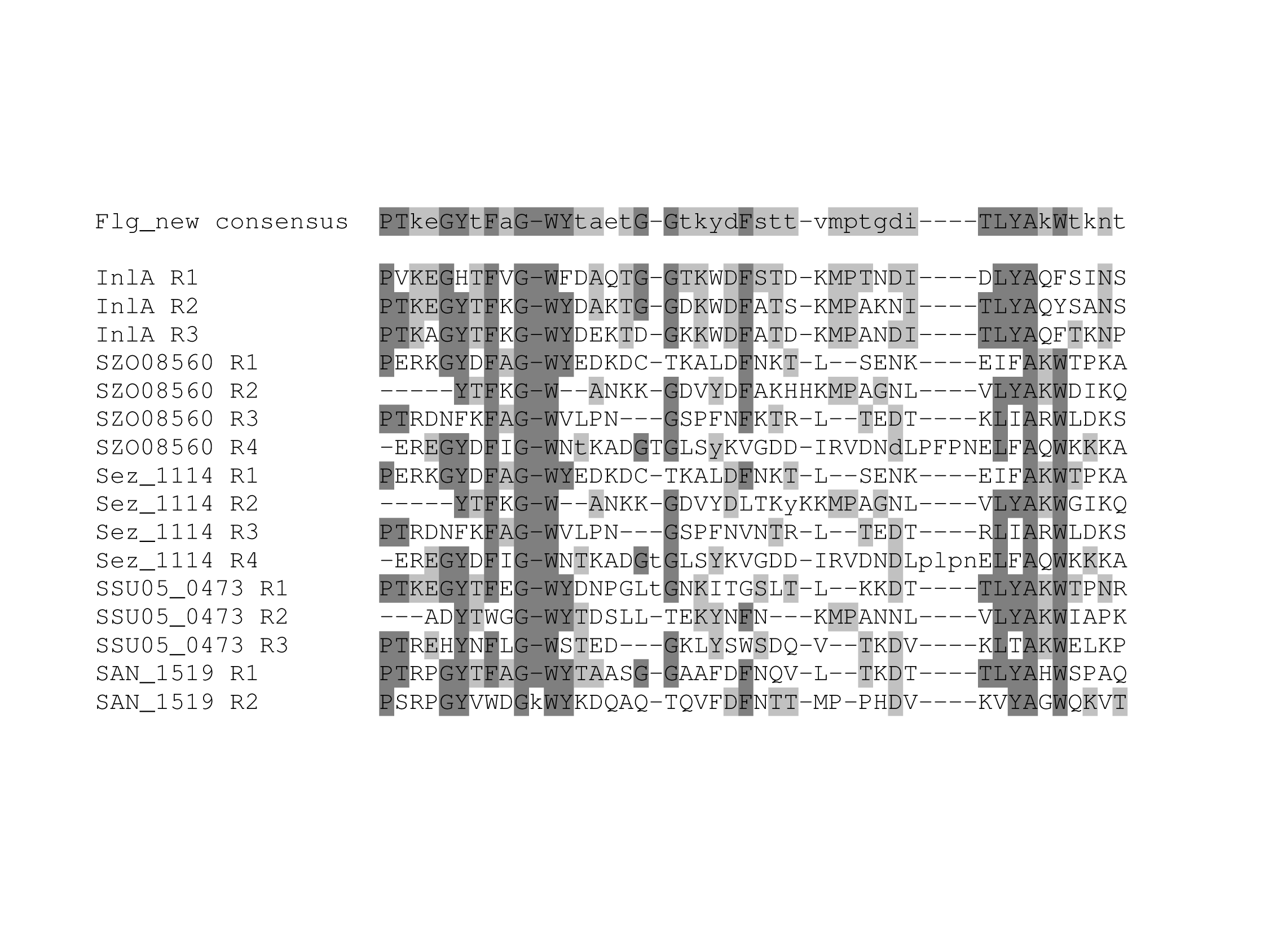

Supplement: Figure S2 — Alignment of SZO08560 and Sez_1114 Listeria-Bacteroides repeat domains domains. Alignment of protein domains in InlA, (Listeria monocytogenes, ABO32414), SZO08560 (SzH70), Sez_1114 (SzMGCS10565), SSU05_0473 (Streptococcus suis strain 05ZYH33, A4VTK0) and SAN_1519 (Streptococcus agalactiae strain COH1, Q3D8T2) to the Pfam hidden Markov model (HMM) for the Listeria-Bacteroides repeat domain (PF09479). Listeria-Bacteroides repeat domains are a feature of some Bacteroides forsythus proteins and families of internalins of Listeria species. Matches to the highly conserved and less well conserved Listeria-Bacteroides repeat domain residues are shown in dark and light grey respectively. (0.70 MB TIF) [file ppat.1000346.s007.tif]
